# International Union of Basic and Clinical Pharmacology. CVII. Structure and Pharmacology of the Apelin Receptor with a Recommendation that Elabela/Toddler Is a Second Endogenous Peptide Ligand

**DOI:** 10.1124/pr.119.017533

**Published:** 2019-10

**Authors:** Cai Read, Duuamene Nyimanu, Thomas L. Williams, David J. Huggins, Petra Sulentic, Robyn G. C. Macrae, Peiran Yang, Robert C. Glen, Janet J. Maguire, Anthony P. Davenport

**Affiliations:** Experimental Medicine and Immunotherapeutics, University of Cambridge, Centre for Clinical Investigation, Addenbrooke’s Hospital, Cambridge, United Kingdom (C.R., D.N., T.L.W., D.J.H., P.S., R.G.C.M., P.Y., J.J.M., A.P.D.); The Centre for Molecular Informatics, Department of Chemistry, University of Cambridge, Cambridge, United Kingdom (D.J.H., R.C.G.); and Computational and Systems Medicine, Department of Surgery and Cancer, Faculty of Medicine, Imperial College London, London, United Kingdom (R.C.G.)

## Abstract

The predicted protein encoded by the APJ gene discovered in 1993 was originally classified as a class A G protein-coupled orphan receptor but was subsequently paired with a novel peptide ligand, apelin-36 in 1998. Substantial research identified a family of shorter peptides activating the apelin receptor, including apelin-17, apelin-13, and [Pyr^1^]apelin-13, with the latter peptide predominating in human plasma and cardiovascular system. A range of pharmacological tools have been developed, including radiolabeled ligands, analogs with improved plasma stability, peptides, and small molecules including biased agonists and antagonists, leading to the recommendation that the APJ gene be renamed APLNR and encode the apelin receptor protein. Recently, a second endogenous ligand has been identified and called Elabela/Toddler, a 54-amino acid peptide originally identified in the genomes of fish and humans but misclassified as noncoding. This precursor is also able to be cleaved to shorter sequences (32, 21, and 11 amino acids), and all are able to activate the apelin receptor and are blocked by apelin receptor antagonists. This review summarizes the pharmacology of these ligands and the apelin receptor, highlights the emerging physiologic and pathophysiological roles in a number of diseases, and recommends that Elabela/Toddler is a second endogenous peptide ligand of the apelin receptor protein.

## I. Introduction

The predicted protein encoded by the APJ gene was discovered by O’Dowd et al. (1993) and was originally classified as a class A G protein-coupled orphan receptor but was subsequently paired with a novel peptide ligand, apelin 36 (APJ endogenous ligand) discovered by Tatemoto et al. (1998). Since then, there has been a large body of work studying the relationship between the ligand and receptor, as well as their physiologic and pathophysiological roles in a number of diseases. Recently, a second proposed endogenous ligand for the apelin receptor has been discovered independently by two groups, and called Elabela (“epiboly late because endoderm late,” which is the first observable phenotype when deleted in zebrafish) by Chng et al. (2013) and Toddler (referring to the loss of motogen properties when deleted) by Pauli et al. (2014). Elabela/Toddler is a 54-amino acid peptide, originally identified in the genomes of fish and humans and misclassified as a non-coding region and hiding in plain sight. It is cleaved to produce a 32 amino acid mature secreted protein (Chng et al., 2013; Pauli et al., 2014). The International Union of Basic and Clinical Pharmacology Committee on Receptor Nomeclature and Drug Classification (NC-IUPHAR) recommends that Elabela/Toddler is a second endogenous ligand for the apelin receptor. Following the convention of naming the peptide according to the precedence of discovery, the nomenclature that is recommended is Elabela/Toddler, abbreviated to ELA (Chng et al., 2013). ELA is an endogenous ligand, functional in the adult mammalian system (Yang et al., 2017b) and is blocked by apelin receptor antagonists. Interestingly, although it shows little sequence homology to apelin with only about 25% conservation (Xie et al., 2014), there is some similarity in the location of hydrophobic residues.

The discovery of this new ligand opens up a number of exciting possibilities. It greatly enhances the spatiotemporal signaling potential through the apelin receptor and how it is modulated in disease, with evidence that ELA, like apelin, is downregulated in human pulmonary arterial hypertension and animal models of the disease already demonstrated (Goetze et al., 2006; Alastalo et al., 2011; Chandra et al., 2011; Kim et al., 2013; Yang et al., 2017b). Meanwhile, it also offers the possibility to explore a new class of ligand at the apelin receptor based on the structure of ELA. However, perhaps most of all, it suggests that, in addition to ELA, there may be other genes of pharmacological importance located in regions of the genome that have previously been overlooked.

This review will discuss the structure and signaling pathways of the apelin receptor and its endogenous ligands, apelin and ELA, before moving on to the development of synthetic agonists and antagonists. It will discuss some of the roles that apelin and ELA have been shown to play in both physiologic and pathophysiological conditions, highlighting the importance of the two ligands and the therapeutic potential of targeting the apelin system.

The following should be consulted for more details of the role of apelin receptor ligands in homeostasis, cell signaling, and aging: Galanth et al. (2012), O’Carroll et al. (2013), Chapman et al. (2014), Flahault et al. (2017), Zhou et al. (2017). The following reviews focus on the apelin signaling pathway in disease: cardiovascular, Scimia et al. (2014), Dalzell et al. (2015), Kuba et al. (2019); myocardial ischemia and reperfusion injury, Chen et al. (2016); vascular smooth muscle, Luo et al. (2018); endothelial cell dysfunction, Cheng et al. (2019); pulmonary hypertension, Kim (2014); hypertension, Gilbert (2017); stroke Wu et al. (2017b); renal, Huang et al. (2018); liver, Lv et al. (2017); cancer, Yang et al. (2016); diabetes and metabolic diseases Castan-Laurell et al. (2012, 2019), Bertrand et al. (2015), Chaves-Almagro et al., (2015), Hu et al., (2016), Alipour et al., (2017).

## II. Recommendations for Nomenclature

The approved Human Genome Organisation (HUGO) Gene Nomenclature Committee (HGNC) symbol for the gene encoding the human apelin receptor is APLNR. While other aliases, including AGTRL1, APJ, APJR, and FLJ90771 exist, it is recommended by NC-IUPHAR that APLNR is used. Similarly, following the identification of apelin, NC-IUPHAR recommend “apelin receptor” as the preferred nomenclature for the receptor protein, adhering to the convention of naming a receptor after its endogenous ligand (Pitkin et al., 2010, Alexander et al., 2017; http://www.guidetopharmacology.org/GRAC/FamilyIntroductionForward?familyId=7). APJ, one of the original names for the gene, also continues to be widely used as a name for the receptor protein. Despite the identification of ELA as a second endogenous ligand at the apelin receptor, it is not recommended that the nomenclature of the receptor is changed. The HGNC have assigned the gene encoding ELA with the symbol “*APELA*” and the name “apelin receptor early endogenous ligand.” Previous gene symbols include *Ende* (mouse, Hassan et al., 2010) and *ELA* (human, Chng et al., 2013). In this review we recommend that the protein encoded by *APELA* be named “ELA,” following the convention of naming by precedence of discovery.

## III. Apelin Receptor Structure

The human apelin receptor has a seven transmembrane structure and consists of 380 amino acids. It was initially identified through homology with the angiotensin II type 1 receptor (AT_1_) with which it shares 54% sequence similarity in the transmembrane domains (O’Dowd et al., 1993). Despite this homology the apelin receptor does not bind angiotensin and, until the recent discovery of ELA, was thought to bind only its cognate ligand apelin. There are no known apelin receptor subtypes in mammals. No binding was detected using 12 apelin peptides ranging from apelin-10 to apelin-36 and 4 precursors screened against 82 human orphan GPCRs (including orphan GPCRs with the highest sequence similarity GPR15 and GPR25) using *β*-arrestin recruitment assays (Southern et al., 2013). The receptor is well conserved with 91% and 89% homology with the 377-amino acid long mouse and rat receptors, respectively. In the zebrafish there are two receptor subtypes, aplnra and aplnrb. Aplnrb shows greater homology with mammalian apelin receptors, and it is in this receptor that the only known naturally occurring mutation occurs, the grinch^s608^ mutation. This mutation involves a Trp^85^ to Leu^85^ amino acid change in the second transmembrane domain and a loss of apelin binding, which in the most severely affected mutants causes a failure of the heart to develop (Scott et al., 2007). It is unknown whether the second zebrafish apelin receptor is a result of duplication or whether mammalian systems have lost this as a vestigial receptor, perhaps offering an explanation for the limited homology between apelin and ELA.

Ma et al. (2017) recently reported the 2.6-Å resolution crystal structure of the human apelin receptor in complex with a synthetic 17-amino-acid apelin analog agonist (PDBID 5VBL). The receptor shows the expected seven transmembrane helical structure with a short eighth helix also observed in other GPCRs. The structure is in reasonable agreement with studies using nuclear magnetic resonance (Langelaan et al., 2013) and molecular dynamics simulations (Macaluso and Glen, 2010; Yang et al., 2017b). In addition, many of the key interfacial receptor-to-agonist contacts identified from the crystal structure are in agreement with mutation data on apelin binding (Table 1). In particular, the evolutionarily conserved residues Arg^168^ and Lys^268^ are predicted to make key contacts with the C terminus of the apelin peptide. However, a number of changes to the receptor and ligand were introduced to achieve crystallization, and it is worth noting the following caveats that prompt a cautious interpretation of the data. Looking at the receptor, residues were removed from the N terminus (residues 1–6) and C terminus (residues 331–380); meanwhile, mutations V117A and W261K were introduced. These mutations force the intracellular portion into an inactive state and render the receptor unable to bind apelin-13. Looking at the ligand, a synthetic 17-amino acid apelin analog agonist was used, which is significantly different from apelin. In particular, a macrocycle and significant mutations have been introduced and these may alter the peptide conformation and its interactions. Finally, the crystal structure is unable to explain the importance of the Arg^2^ and Leu^5^ residues of apelin 13, known key binding elements from mutation data (Fan et al., 2003; Medhurst et al., 2003).

**TABLE 1:** Contacts between apelin-13 and the apelin receptor, inferred from the crystal structure PDBID 5VBL

Apelin-13 Contact	Receptor Contacts	References
Q1 Sidechain	D92*^a^*	Gerbier et al., 2015
Q1 Backbone	N177	
P3 Sidechain	T22	
	D23*^a^*	Zhou et al., 2003a
R4 Backbone	D23*^a^*	Zhou et al., 2003a
L5 Sidechain	E20*^a^*	Zhou et al., 2003a
	T22	
S6 Sidechain	E174*^a^*	Chapman et al., 2014; Gerbier et al., 2015
S6 Backbone	Y21	
H7 Sidechain	E174*^a^*	Chapman et al., 2014; Gerbier et al., 2015
K8 Sidechain	Y21	Gerbier et al., 2015
	D284*^a^*	Ma et al., 2017
	S275	
G9 backbone	Y271	
P10 Sidechain	E198*^a^*	
	L173	Chapman et al., 2014
M11 Sidechain	W24	
	Y271*^a^*	Ma et al., 2017
	F291	
	K268*^b^*	Kumar et al., 2016
P12 Sidechain	W24	
	Y93	
P12 Backbone	R168*^a^*	Ma et al., 2017
F13 Sidechain	W85*^a^*	
	Y88	
	T89	
	Y93	
	Y299	
F13 Carboxylate	K268*^b^*	Kumar et al., 2016
	Y264	

^a^ Receptor residues implicated in apelin binding by mutagenesis.

^b^ Receptor residues affecting bias and internalization by mutagenesis.

**Fig. 1.**
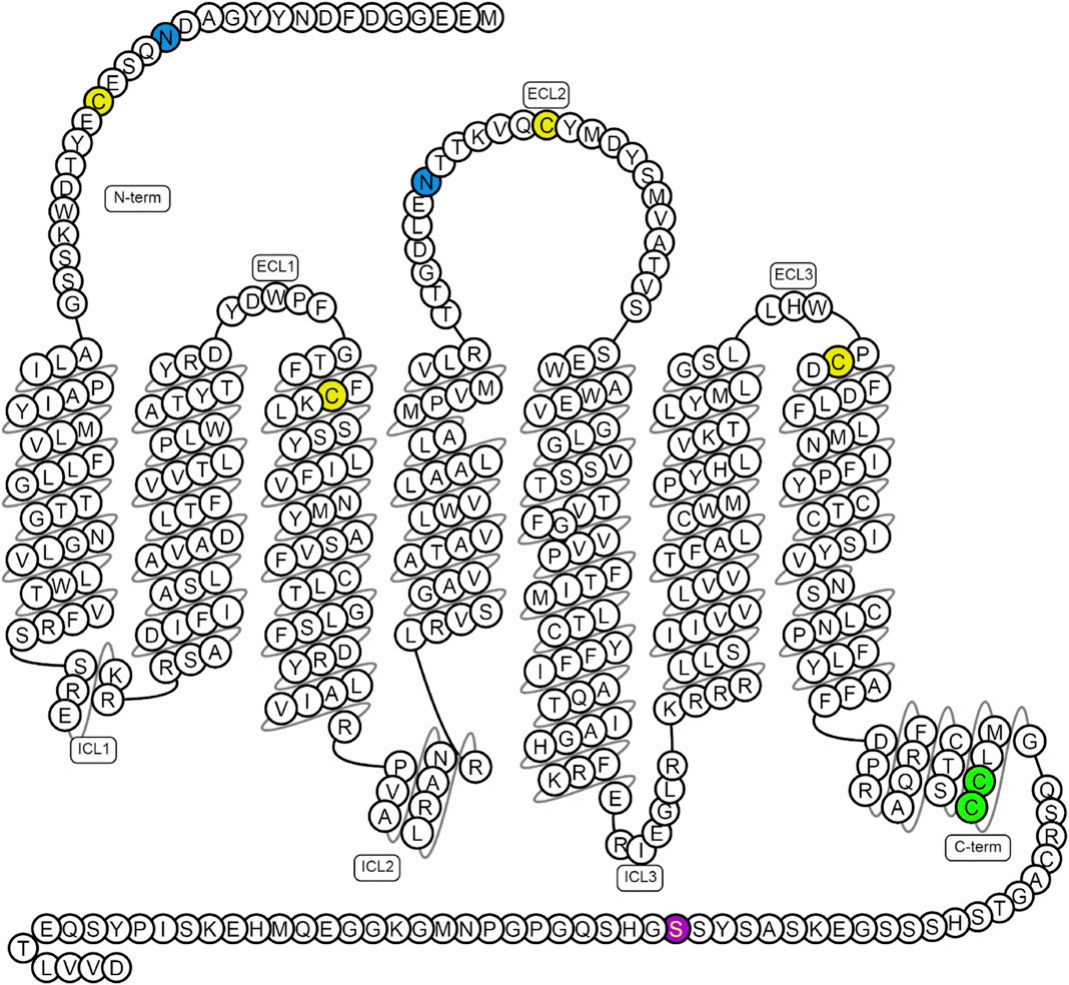
Predicted disulfide bridges are between Cys^19^–Cys^281^ and Cys^102^–Cys^181^ (yellow); glycosylation sites (blue) are in the N-terminal tail (Asn^15^) and extracellular loop 2 (ECL2; Asn^175^); palmitoylation site (green) Cys^325^ and Cys^326^ and phosphorylation site (purple) Ser^348^ have been confirmed experimentally, of which Ser^348^ is crucial for apelin receptor interactions with GRK2/5, *β*-arrestin, and its internalization (Chen et al., 2014). Figure constructed from G protein-coupled receptor database (Pándy-Szekeres et al., 2018).

Although the structure confirms the presence of two disulfide linkages present in a number of class A GPCRs (Cys^19^-Cys^281^ and Cys^102^-Cys^181^) (Fig. 1), it does not clarify the importance of posttranslational modifications in the apelin receptor. The receptor is likely to be glycosylated (Medhurst et al., 2003) and contains two glycosylation motifs expected at residues in the N-terminal tail (Asn^15^) and extracellular loop 2 (Asn^175^), both of which appear to be involved in agonist binding. Glycosylation is known to be significant in GPCR function (Lanctot et al., 1999) and, in some cases, affects agonism and bias (Soto et al., 2015), but its importance in the case of the apelin receptor is not known. In addition, there are a number of potential palmitoylation sites in the intracellular C-terminal region of the receptor. Based on predictions from the SwissPalm protein S-palmitoylation database (Blanc et al., 2015) and CSS-Palm (Ren et al., 2008), residues Cys^325^ and Cys^326^ on the putative helix TM8 are expected to be palmitoylated. Palmitoylation is key in GPCR expression and function (Qanbar and Bouvier, 2003). Finally, the C terminus appears to contain a “phosphorylation barcode” (Nobles et al., 2011) and Ser^348^ has been identified as a novel phospho-regulatory site (Chen et al., 2014) (Fig. 1).

## IV. Apelin Receptor Signaling in the Cardiovascular System

Infusion of apelin leads to vasodilatation in humans in vitro (Maguire et al., 2009) and in vivo (Brame et al., 2015) and in rodents in vivo (Tatemoto et al., 2001). The second main cardiovascular action is positive cardiac inotropy in vitro (Szokodi et al., 2002; Maguire et al., 2009; Perjés et al., 2014) and in vivo in rats (Berry et al., 2004; Jia et al., 2006; Atluri et al., 2007), mice (Ashley et al., 2005), and humans (Japp et al., 2008, 2010; Barnes et al., 2013) without hypertrophy. Interestingly, in denuded vessels, apelin promotes vasoconstriction (Katugampola et al., 2001; Maguire et al., 2009) and it is only in intact tissues that the endothelium-dependent vasodilatation is observed. This vasodilatation has been suggested to be either nitric oxide dependent (Tatemoto et al., 2001) or prostanoid dependent (Maguire et al., 2009), perhaps reflecting species or whole organism versus isolated vessel differences.

**Fig. 2.**
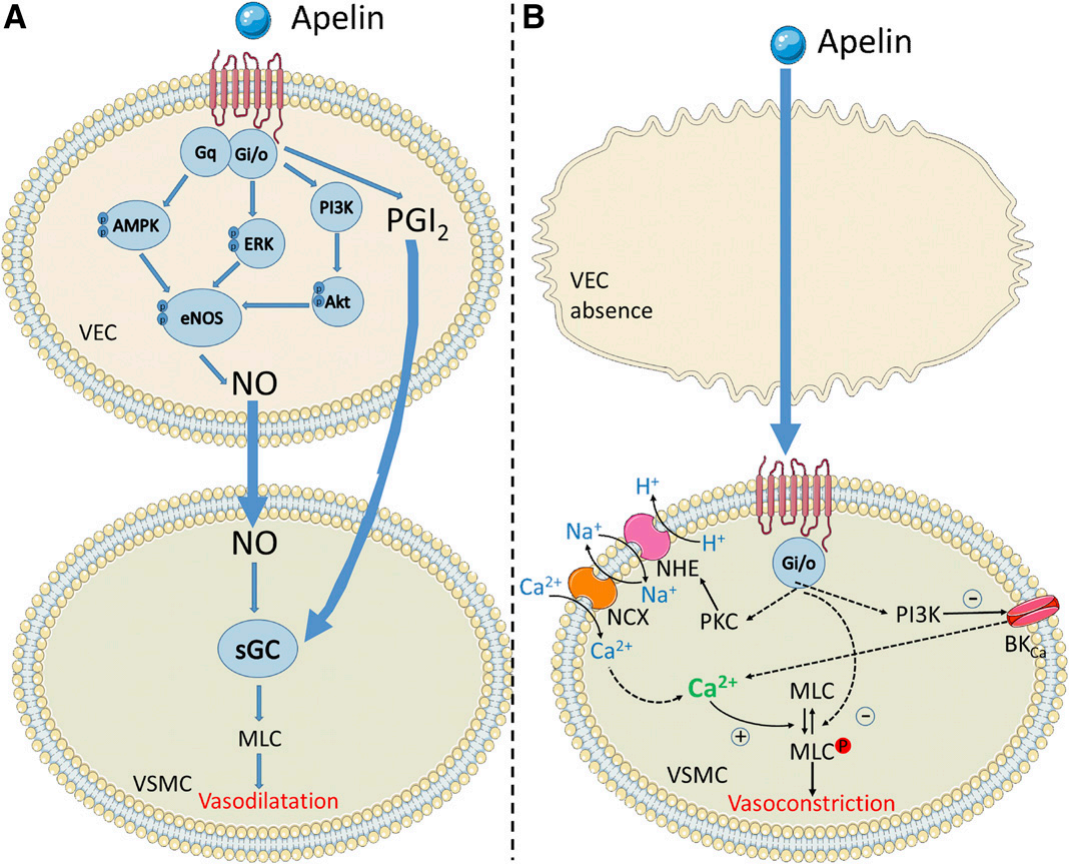
The key signaling pathways suspected to be activated in vascular endothelial cells (VEC) and smooth muscle cells (VSMC) by the apelin receptor. Apelin binding can promote G*α*i, G*α*q, and *β*-arrestin recruitment to the receptor. In the presence of the endothelium, both G*α*i and G*α*q promote relaxation of smooth muscle cells through nitric oxide and prostacyclin release. In the absence of the endothelium, apelin binds directly to the receptor on the smooth muscle cells and leads to constriction through undetermined intermediate steps but most likely involving PKC, phosphoinositide 3-kinase (PI3K) and myosin light chain phosphorylation. Figure constructed using Servier Medical Art.

Mechanistically, these processes are poorly understood, although the apelin receptor is thought to signal primarily through G_*α*i_, leading to decreased intracellular cyclic adenosine monophosphate (cAMP) by inhibition of adenylyl cyclase, as evidenced by inhibition of forskolin-stimulated cAMP production (Habata et al., 1999) and pertussis toxin sensitivity (Hosoya et al., 2000; Masri et al., 2002). Figure 2 shows a summary of the possible downstream pathways activated following G_*α*i_ activation by the apelin receptor as suggested by Yang et al. (2015a).

**Fig. 3.**
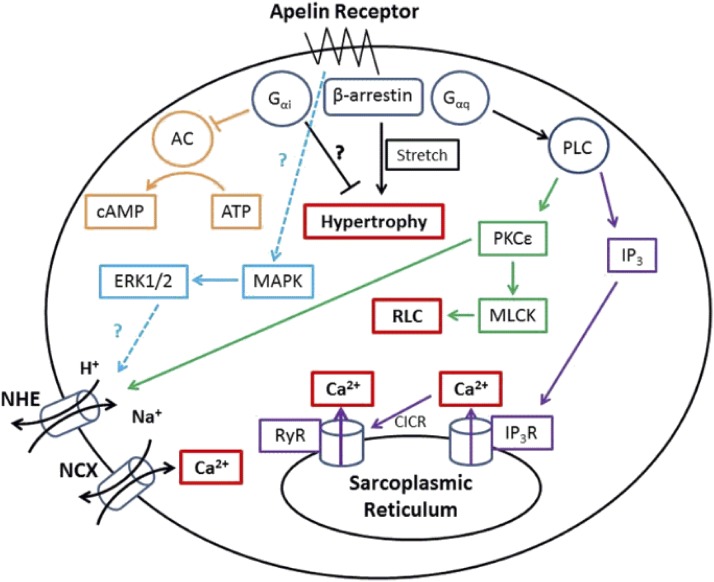
The key signaling pathways suspected to be activated in cardiomyocytes by the apelin receptor. Apelin binding can promote G_*α*i_, G_*α*q_, and *β*-arrestin recruitment to the receptor, these pathways are thought to ultimately lead to cardiac inotropy without hypertrophy. However, in the absence of apelin, *β*-arrestin recruitment may lead to stretch-mediated hypertrophy.

As well as activating G_*α*i_, there is also significant evidence that the apelin receptor may couple to G_*α*q_, particularly in cardiomyocytes (Szokodi et al., 2002), promoting phospholipase C and, in turn, inositol triphosphate (IP_3_) and protein kinase C*ε* activity (PKC*ε*, but not PKC*α*; Perjés et al., 2014). IP_3_ activates IP_3_ receptors and Ca^2+^ release, which can feed back to ryanodine receptors, leading to calcium-induced calcium release. PKC*ε* might enhance Na^+^-H^+^ exchange on the sarcolemma, increasing intracellular Na^+^ and consequently enabling the Na^+^-Ca^2+^ exchanger to raise intracellular Ca^2+^ concentrations (Chandrasekaran et al., 2008). It has also been suggested that extracellular signal regulated kinases 1/2 can regulate cardiac contractility through an independent mechanism (Perjés et al., 2014), although how this pathway is activated or increases contractility is not clear. Finally, it has also been suggested that apelin can activate myosin light chain kinase, which can phosphorylate the regulatory light chain of myosin II, resulting in greater Ca^2+^ sensitivity of the force generating machinery (Perjés et al., 2014). These pathways are summarized in Fig. 3.

## V. Endogenous Agonists

**Fig. 4.**
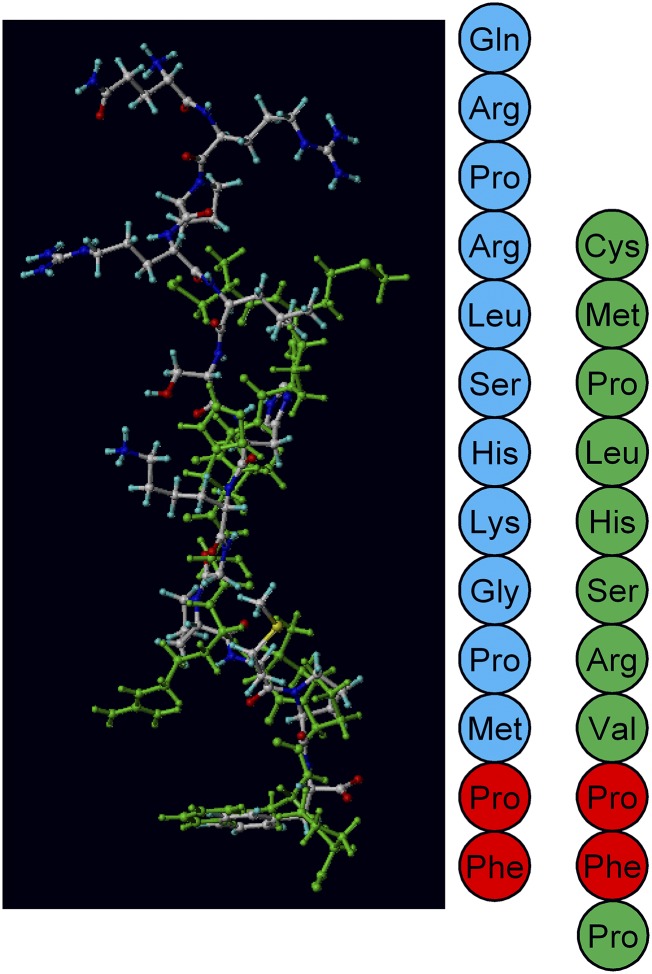
An overlay of ELA-11 (green) and apelin-13 (blue) docked in the apelin receptor binding pocket. The peptide sequences are shown alongside with the same color scheme. The red amino acids show where identical residues line up. Overlay from Yang et al., (2017b) under CC-BY license.

To date, two endogenous peptide agonists have been identified at the apelin receptor, apelin and ELA. Although they show limited sequence homology, they have similarity in the location of hydrophobic residues and can be docked into the same binding pocket in a molecular docking/dynamic simulation of the receptor (Fig. 4). Apelin was discovered in bovine stomach extracts by Tatemoto et al. (1998), and since then there has been a significant advance in our understanding of the isoforms of apelin and their binding to the apelin receptor. ELA, however, was only discovered relatively recently by two groups working independently (Chng et al., 2013; Pauli et al., 2014), and as such, there is still much to be learned about its endogenous isoforms and interactions at the apelin receptor. The important question of why there are two endogenous ligands for the apelin receptor and their relationship remains to be addressed. Intriguingly, pharmacological studies suggest that ELA-11 may display some bias toward the G protein pathway versus *β*-arrestin, and it can be speculated that this peptide might function as a biased endogenous ligand (Yang et al., 2017b).

### A. Apelin

**Fig. 5.**
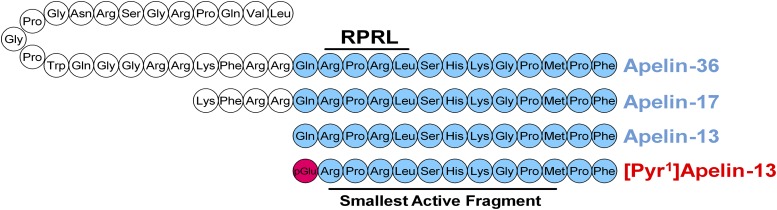
The amino acid sequences of cleaved apelin fragments. [Pyr^1^]apelin-13 is the predominant form in the cardiovascular system and is shown in red with the pyroglutamate residue in pink. The smallest active fragment is highlighted, as well as the RPRL motif which has been thought critical to binding.

Apelin is expressed as a 77-amino acid pre-protein, pre-pro-apelin, consisting of the 55-amino acid pro-apelin fragment and an N-terminal secretory sequence. Following secretion, pro-apelin is cleaved to produce three main apelin fragments, apelin-36, apelin-17, and apelin-13, the last of which can undergo cyclization of the glutamine at its N terminus to produce pyroglutamated apelin-13 ([Pyr^1^]apelin-13; Fig. 5) (Pitkin et al., 2010). [Pyr^1^]apelin-13 has been shown to be the predominant isoform in the human cardiovascular system (Maguire et al., 2009) and human plasma (Zhen et al., 2013) using mass spectrometry to distinguish the isoforms. In plasma from human volunteers, endogenous apelin-36, apelin-17, and apelin-13 were not detected (Zhen et al., 2013).

It was originally hypothesized that cleavage occurred sequentially with pro-apelin first cleaved to apelin-36 and then in turn to the smaller apelin-17 and apelin-13 fragments, always retaining the bioactive C terminus. These cleavages were predicted due to expected basic cleavage sites in the peptide sequence and the identification of apelin-36, -17, and -13 as active sequences found in biologic samples (Kleinz and Davenport, 2005). However, because of this, the enzymes involved are not well characterized and warrant further investigation. This is especially true for the production of apelin-36 and -17. Recently, Shin et al. (2013) proposed that furin can directly cleave pro-apelin to apelin-13 in vitro without producing these longer isoforms. Apelin-36 is also cleaved by angiotensin converting enzyme 2 (ACE2) with high catalytic efficiency to remove the C-terminal phenylalanine. Similarly, apelin-13 can be cleaved by ACE2 to produce apelin-13_(1–12)_ (Vickers et al., 2002). Interestingly, apelin-13_(1–12)_ retains activity, as demonstrated by recruitment of *β*-arrestin to the apelin receptor in vitro, constriction of saphenous vein ex vivo, and positive inotropy and blood pressure decrease in vivo. However, another report has shown that ACE2 cleavage of [Pyr^1^]apelin-13 and apelin-17 abolished the cardioprotective effects of these peptides in a myocardial ischemia-reperfusion mouse model (Wang et al., 2016). Most critically, apelin-13_(1–12)_ was able to induce an increase in forearm blood-flow in human volunteers (Yang et al., 2017a). Additional support for this is evidenced by the retention of activity when the terminal phenylalanine of apelin-13 is mutated to alanine (Fan et al., 2003; Medhurst et al., 2003; Yang et al., 2017a). Furthermore, C-terminal truncation studies have suggested activity is retained even as far as apelin-13_(1–11)_ (Zhang et al., 2014). This last study also reported that the N-terminal glutamine in position 1 is not essential for binding either, suggesting that the 10-amino acid apelin-13_(2–11)_ fragment is the smallest active fragment (Zhang et al., 2014). Although the N-terminal glutamine residue is not essential for binding, the cyclization to pyroglutamate is widely observed in vivo and enhances the stability of the peptide fragment (Van Coillie et al., 1998; Habata et al., 1999). Interestingly, while the C-terminal amino acids of apelin-36 make the smallest active fragment for binding to the apelin receptor, it has been suggested that N-terminal portions may aid the molecule in interacting with the receptor (Hosoya et al., 2000).

Having considered the smallest active fragments of apelin, it is important to discuss how the different fragments bind to the receptor and maintain activity to inform the development of synthetic molecules. Deductions of apelin binding have been largely achieved through alanine scanning mutagenesis and receptor modeling approaches; a crystal structure was only very recently reported (Ma et al., 2017). It was initially found that mutating the arginine residues in positions 2 and 4 of the apelin peptide greatly reduced the ability of the fragment to bind to the receptor (Fan et al., 2003; Medhurst et al., 2003). This, alongside the detrimental effect of mutating the lysine in position 8, suggested the importance of a positive charge on the apelin peptide for interacting with the receptor (Fan et al., 2003). Concurrent studies utilizing the receptor determined that the second 10 N-terminal residues of the apelin receptor are essential to interact with the ligand. These residues are largely negatively charged, with the N terminus of the apelin receptor possessing a net charge of −7, supporting the hypothesis that ionic interactions play a critical role in apelin binding to its receptor (Zhou et al., 2003a). Meanwhile, Medhurst and colleagues (2003) reported that the largest loss of binding observed in their alanine mutagenesis studies occurred with loss of leucine in position 5 and postulated that the “RPRL” motif from positions 2 to 5 plays a critical role in apelin binding. Macaluso and Glen (2010) confirmed the importance of the RPRL motif through elegant cyclization experiments. They showed that the RPRL sequence produces a favorable *β*-turn motif, which is essential in interaction with the apelin receptor. When they locked the peptide in an unfavorable turn at the RLSH sequence, these cyclized peptides were unable to bind the receptor efficiently despite higher sequence homology to the endogenous agonist.

Recent studies have found support for both ionic and RPRL sequence interactions and propose a two-step binding process for apelin peptides to the receptor. Initial ionic interactions occurring between the N-terminal tail of the receptor and the ligand promote binding of the ligand to the receptor. Following this, the ligand moves deeper into the binding pocket where it is able to form more stable interactions and promote receptor activation (Iturrioz et al., 2010b; Langelaan et al., 2013; Gerbier et al., 2015). This greater understanding of how apelin peptides bind to the receptor has informed the design of a number of synthetic agonists based on the apelin sequence and these will be discussed in section VIII.

In addition, isoforms comprising various lengths of amino acids may activate different downstream pathways at the receptor, leading to different signaling bias. It is important to understand this as any differences could be exploited physiologically by enhanced production of one isoform in a given tissue or at a given time. Additionally, the endogenous bias observed could be used to inform the development of synthetic biased molecules. One area that has been explored is the rate of internalization and recycling of receptor-ligand complexes, which appears to be highly ligand dependent (Zhou et al., 2003a; Lee et al., 2010). Lee et al. (2010) demonstrated that apelin-13-mediated internalization could be rapidly reversed when washed out, whereas apelin-36 resulted in more prolonged receptor internalization. El Messari et al. (2004) have also reported that apelin-17 is better able to recruit *β*-arrestin and internalize the receptor than apelin-13. Meanwhile, it has been shown that loss of the C-terminal phenylalanine can induce bias toward G protein signaling (Ceraudo et al., 2014). These studies, therefore, suggest that longer length peptides are able to reach a binding pocket that is not accessible to the shorter apelin-13 isoform to induce *β*-arrestin recruitment and internalization. This is further supported by longer length ELA peptides also possessing *β*-arrestin bias (Yang et al., 2017b). Accessing this deeper pocket in the receptor likely leads ultimately to phosphorylation of the C-terminal Ser^348^ residue that, when mutated, showed abolition of the G protein receptor kinase/*β*-arrestin pathway signaling, while preserving signaling through the G protein pathway (Chen et al., 2014). It is interesting that *β*-arrestin recruitment and internalization of the apelin receptor appear to be mediated by only one phosphorylation site and not by a more complex “phosphorylation barcode” as suggested for other GPCRs (Tobin et al., 2008a; Butcher et al., 2011). Furthermore, it has been suggested that the *β*-arrestins are not internalized with the ligand-receptor complex, unlike for some other GPCRs (Evans et al., 2001).

Although as discussed above, apelin-13 is cleaved by ACE2, to the product apelin-13(_1–12_) that retains biologic activity (Vickers et al., 2002). The metalloprotease neprilysin has been shown to metabolize [Pyr^1^]apelin-13 between Arg^4^ and Leu^5^ and also between Leu^5^ and Ser^6^ with the C‐terminal fragments 5–13 and 6–13 accumulating as a result of this activity. These fragments did not bind to the apelin receptor, thereby making neprilysin the first protease to be identified to fully inactivate apelin (McKinnie et al., 2016). Neprilysin inhibitors such as the pro-drug sacubitril are used in heart failure (Velazquez et al., 2019). These results suggest an additional benefit would be to reduce apelin inactivation to cause a beneficial vasodilatation and increase in cardiac output. This hypothesis has not yet been tested in patients being treated with sacubitril.

### B. Elabela/Toddler

The endogenous agonist, ELA, was named following its discovery in 2013 by Chng et al. (2013). It was also independently given the name “Toddler” after its identification as a motogen during gastrulation (Pauli et al., 2014). Previously, the gene encoding the peptides was named *Ende* when studied as a novel transcript involved in the development of mouse endoderm (Hassan et al., 2010). Since then the gene encoding the peptide has been renamed *APELA* (apelin receptor early endogenous ligand) by the HUGO Gene Nomenclature Committee (HGNC, section II).

**Fig. 6.**
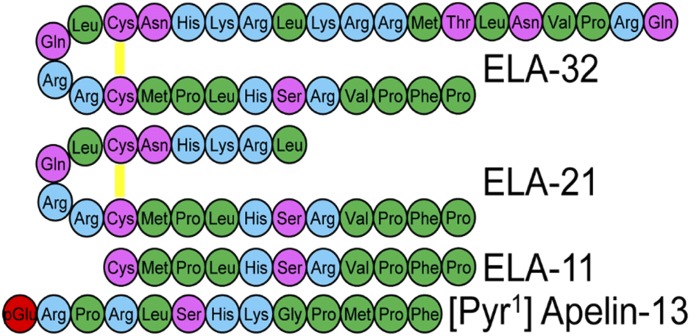
The amino acid sequences of the predicted cleaved ELA fragments compared with [Pyr^1^]apelin-13, the predominant apelin isoform in the cardiovascular system. There is little sequence homology between ELA and apelin fragments; however, there are some similarities in the positioning of charged residues. Disulfide bridges are yellow lines, hydrophobic amino acids are shown in green, uncharged polar amino acids in pink, basic amino acids in blue and pyroglutamate in red. From Yang et al. (2017b) under CC-BY license.

Interestingly, ELA was identified in a previously designated noncoding region of the genome, but the existence of the peptide had already been predicted owing to discrepancies between apelin and apelin receptor mutations (Charo et al., 2009). Notably, knock out of the apelin receptor in mice caused prenatal mortality (Ishida et al., 2004; Charo et al., 2009; Roberts et al., 2009; Scimia et al., 2012; Kang et al., 2013) due to disrupted cardiac development with rudimentary to no heart (Kang et al., 2013). In contrast, apelin knockouts had normal heart development (Kidoya et al., 2008; Charo et al., 2009), although they were at greater risk from age-related and induced disease (Kuba et al., 2007). Studies exploring ELA knockouts in zebrafish found that these phenocopied the apelin receptor mutations (Chng et al., 2013; Pauli et al., 2014). Furthermore, it has been shown that during development the apelin receptor is expressed during gastrulation at the same time as ELA, whereas apelin is not expressed until the end of gastrulation (Scott et al., 2007; Pauli et al., 2014). While this ligand was first discovered in zebrafish embryos as a factor involved in cardiac development, it has since been shown to have activity in adult mammalian systems (Murza et al., 2016; Perjés et al., 2016; Yang et al., 2017b) and its expression is altered in disease (Yang et al., 2017b). During its discovery in zebrafish development, ELA was found to be a 54-amino acid peptide that was cleaved to produce a 32-amino acid mature secreted protein. This mature protein was in turn predicted to undergo cleavage by furin to produce two more fragments of length, 21 and 11 amino acids (Chng et al., 2013; Pauli et al., 2014), all of which have been pharmacologically characterized by Yang et al. (2017b); Fig. 6. Murza et al. (2016) have also suggested fragments of 22 and 14 amino acids with a potential 23-amino acid variant. It is notable that the 11-amino acid fragment is invariant between species, suggesting strong evolutionary conservation. Interestingly, ELA is more strongly positively charged than apelin and also displays higher binding affinities to the receptor at corresponding fragment sizes, lending support to an ionic interaction as critical to binding.

## VI. Apelin Receptor Distribution

### A. Distribution of the Apelin Receptor in Human Tissues

Several studies have investigated the distribution of the apelin receptor in human tissues (Table 2). The apelin receptor is widely expressed in all human tissues receiving a blood supply, consistent with expression on endothelial cells that line every blood vessel and mediate vasodilatation. Apelin receptors are also expressed on other cell types such as cardiomyocytes to mediate positive inotropy (Kleinz et al., 2005). Expression of the mRNA has been observed in all regions of the brain, such as the cortex (including the hippocampus and amygdala), subcortical regions (including the hypothalamus, nucleus accumbens, thalamus, caudate nucleus, putamen), midbrain (including substantia nigra), hindbrain (including cerebellum and medulla oblongata), pituitary, and spinal cord (Medhurst et al., 2003). The highest levels were reported in the corpus callosum and spinal cord; however, using Northern blotting, other studies reported abundant expression in the brain medulla, amygdala, hippocampus, substantia nigra, hypothalamus, and thalamus, with lower levels in the striatum and cerebral cortex (Matsumoto et al., 1996; Edinger et al., 1998). They also observed high levels in the white and gray matter of the spinal cord. While the distribution of the receptor mRNA in the brain has been studied extensively, the distribution of the receptor protein in the brain has not been reported to date. The apelin signaling pathway regulates fluid homeostasis via hypothalamic-pituitary-adrenal axis as well as modulating cardiovascular function in the forebrain and brain stem (O’Carroll et al., 2013). The precise function of apelin and its receptors in other regions of the central nervous system are still to be explored.

**TABLE 2:** Expression of apelin receptor (APLNR), apelin, and ELA in mouse, rat, and human tissues Compiled from Edinger et al. (1998), Hosoya et al. (2000), Lee et al. (2000), O’Carroll et al. (2000), Medhurst et al. (2003), Kleinz and Davenport (2005), Regard et al. (2008), Pope et al. (2012), Deng et al. (2015), Wang et al. (2015b).

	APLNR			Apelin			ELA		
	Human	Rat	Mouse	Human	Rat	Mouse	Human	Rat	Mouse
Brain	+++	++	+	++	+	+++			
-paraventricular nucleus		+	++						
-supraoptic nucleus		++	++						
Anterior pituitary	+*^a^*	+++	+++	++*^a^*	+*^a^*				
Intermediate pituitary		−/+	−/+		+/+++				
Posterior pituitary		−/+	−/++		+/++				
Thymus	+	−	+			−			
Spinal cord	+	+++	++	++	++				
Cerebellum	+	+	−	+	+	−			
Hippocampus	+	+	−	+	+				
Thalamus	+	+		+	+				
Heart	+	++	+++	+	++	++	−/+	+	
Adrenal gland		+	+		+				
Lungs	++	+++	++	+	+++	++	+		
Stomach	+	+	+	−	+				
Liver	+	−	+	−	−	−			
Small intestine	++	+	+	−	+				
Large intestine	++	+	+		+				
Pancreas	+	−	+	+					
Kidney	+	+	+	+	+	+	++	+++	
Testis	+	+	+	+	+	++			
Prostate	+			+			++		
Ovary	+	+	++		+	+			
Uterus	+	+	++	−	+	−			
Placenta	++	++		++					
Mammary gland		+			++				
Skeletal muscle	+	++	++	−	+	+			
Adipose tissue	++	++	++*^b^*		++				
Cartilage	++	++							
Spleen	+++	−	+		−	+			
Skin		+	+						
Bladder		+	+						
Gall bladder			+						

+++, high expression; ++, moderate expression; low expression; −, absence mRNA expression.

^a^ Expression in whole pituitary gland.

^b^ Expression reported in both white and brown adipose tissue.

In the periphery, the highest apelin receptor mRNA expression was reported in the spleen and placenta, with lower levels in the heart, liver, lung, kidney, pancreas, small intestine, stomach, and uterus (Medhurst et al., 2003). No apelin receptor mRNA was reported in skeletal muscle, testes, or prostate. In neonatal tissues, the highest expression was observed in lung, heart, and kidney, with lower levels detected in the small intestine, stomach, spleen, and brain (Hosoya et al., 2000). Apelin receptor is expressed at the protein level in the right and left ventricle of the heart, media, and intima of muscular arteries and large elastic arteries and veins. However, in the lungs, expression was predominantly localized to vascular beds (Katugampola et al., 2001; Kleinz et al., 2005). In addition, expression has been shown in vascular smooth muscle cells and endothelial cells of human kidney and cultured human endothelial cells (Kleinz et al., 2005).

### B. Distribution of the Apelin Receptor in Rat Tissues

The distribution of the apelin receptor in rat tissues has also been extensively studied. O’Dowd et al. (1993) first showed by Northern blotting apelin receptor expression in the hippocampus, thalamus, cortex, and cerebellum. Other studies have reported a similarly wide tissue distribution in the rat compared with humans. The mRNA was detected in the cortex (including hippocampus), subcortical regions (nucleus accumbens, striatum, hypothalamus), midbrain, cerebellum, and pituitary (De Mota et al., 2000; Hosoya et al., 2000; Lee et al., 2000; O’Carroll et al., 2000; Kawamata et al., 2001; Medhurst et al., 2003). Similarly, high apelin receptor expression was observed in the paraventricular nucleus and supraoptic nucleus of the hypothalamus, where it colocalized with vasopressin and oxytocin in magnocellular neurons (O’Carroll and Lolait, 2003; De Mota et al., 2004; Reaux-Le Goazigo et al., 2004). This may support previously identified roles in the control of fluid homeostasis (Reaux et al., 2001; Roberts et al., 2009; Hus-Citharel et al., 2014) and perhaps an as yet unidentified role in reproduction. At the protein level, the expression of the apelin receptor in several regions of the brain has been reported, indicating a good correlation between transcription and translation. Apelin receptor immunoreactivity was observed in both neuronal and glial cells in the rat brain (Medhurst et al., 2003), while [Pyr^1^]apelin-13 binding sites were found in the cerebellum, the basal surface of the hypothalamic diencephalon, paraventricular nucleus (magnocellular and parvocellular neurons), and dorsal surface of the thalamus (Katugampola et al., 2001; Hazell et al., 2012). In the pituitary, one study observed strong expression in the anterior and intermediate lobes (De Mota et al., 2000), while another reported only moderate expression in the anterior pituitary but negligible levels in the intermediate lobe (O’Carroll et al., 2000) and posterior lobe (Pope et al., 2012). This anterior expression is supported in additional studies (Tobin et al., 2008b), although this study did not investigate expression in the intermediate or posterior lobe.

In peripheral tissues, the highest mRNA expression was found in the lung and heart. Other tissues including the kidney, skeletal muscle, placenta, thyroid gland, ovary, uterus, and adipose tissues also show expression (Hosoya et al., 2000; O’Carroll et al., 2000; Medhurst et al., 2003). A high density of the protein is found in the lungs and heart, with lower levels in the kidney cortex (Katugampola et al., 2001).

### C. Distribution of the Apelin Receptor in Mouse Tissues

Receptor expression in mice has been poorly studied. In the mouse brain, low transcript levels are found in the whole brain, cerebellum, hypothalamus, hippocampus, and olfactory bulb (Medhurst et al., 2003; Regard et al., 2008). However, another study using in situ hybridization reported a restricted central distribution in the brain, with strong expression noted in the paraventricular and supraoptic nucleus of the hypothalamus, as well as in the anterior pituitary, but lower levels in the posterior pituitary (Pope et al., 2012). Peripherally, the highest expression levels were observed in the heart, followed by moderate levels in the liver, kidney, lung, skeletal muscle, and spleen, with lowest levels in the testes, thymus, bladder, and ovary (Medhurst et al., 2003; Pope et al., 2012).

### D. Species Differences in Apelin Receptor Distribution

Although the central distribution of the receptor in humans and rats is very similar, this is not the case for the peripheral distribution. While the receptor was not detected in the rat spleen and liver, its expression was observed in these organs in humans as well as mice (Medhurst et al., 2003). This suggests that there could be a species difference in the distribution of the apelin receptor in humans, rats, and mice. Indeed, such species differences were reported by Pope et al. (2012), who characterized the distribution of the receptor in mouse using in situ hybridization and autoradiography. They observed a restricted central distribution of the receptor, with receptor transcript and protein levels abundant in the hypothalamus and anterior pituitary. Rats had a more broad distribution in the central nervous system (Pope et al., 2012). The functional significance of such species difference is currently unknown and may warrant further investigation. It could partly reflect the complexity of the apelin signaling system or that the distribution was limited to regions in the hypothalamic-pituitary-adrenal axis of the mouse brain. Additionally, although Medhurst et al. (2003), using real time polymerase chain reaction, detected low levels of the receptor in mouse liver and testes, Pope et al. (2012) did not detect apelin receptor in these organs using in situ hybridization and autoradiography. This discrepancy reflects the need for more studies to clarify the distribution of the apelin receptor in mouse tissues to correctly infer functions.

## VII. Endogenous Peptide Distribution

### A. Apelin Distribution in Human Tissues

Apelin shares a similar wide distribution with its cognate receptor in the brain and peripheral tissues. In the brain, strong apelin expression was observed in all regions, including the cortex, subcortex, and midbrain as well as pituitary and spinal cord (De Mota et al., 2000; Lee et al., 2000; Medhurst et al., 2003). In the periphery, the highest expression was reported in the placenta, with moderate expression levels in the heart, lung, kidney, and testes, although lower levels were detected in the liver, skeletal muscle, pancreas, spleen, small intestine, and uterus (Habata et al., 1999; Lee et al., 2000; Kawamata et al., 2001; Medhurst et al., 2003). The peptide is also expressed in large conduit vessels, including coronary artery and saphenous vein, renal blood vessels, blood vessels of the adrenal gland, and cells of the cardiac atria and ventricles (Kleinz and Davenport, 2004). Hence, the expression of apelin receptor (see section VI) in organs that do not express the ligand, such as the stomach and liver, may suggest an endocrine function of the ligand where the secreted ligand is transported in circulation to distant sites of action.

### B. Apelin Distribution in Rat Tissues

Apelin has a similar but more widespread distribution in the rat brain compared with the apelin receptor, with high levels of apelin mRNA also expressed in the claustrum, anterior and posterior cingulate, retrosplenial area, olfactory tubercle, and several areas of the thalamic nuclei, including anterodorsal, mediodorsal, ventroposterior, and habenular nuclei (Lee et al., 2000). However, the possibility that apelin receptor may be expressed in some of these regions cannot be excluded, because compared with other studies on the distribution of apelin and its receptor in the brain, Lee et al., (2000) provided the most detailed anatomic distribution. At the protein level, the most detailed characterization of apelin expression in the brain was reported by Reaux et al. (2002). They reported peptide distribution in several brain regions such as telencephalon (including septum, amygdala), diencephalon (including thalamus, preoptic region, hypothalamus), mesencephalon (including gray matter, dorsal raphe, cuneiform nucleus), pons (including dorsal tegmental nucleus, parabrachial nucleus, nucleus of Barrington), medulla oblongata (including spinal trigeminal nucleus, lateral reticular nucleus, nucleus of solitary tract), and circumventricular organs, including subfornical organ, subcommissural organ, and area postrema. Of these regions, the hypothalamus had the highest density of apelin-positive cell bodies and nerve fibers. Other studies confirmed this distribution and found high expression of the peptide, as well as colocalization of apelin immunoreactivity with that of vasopressin (Brailoiu et al., 2002; De Mota et al., 2004; Reaux-Le Goazigo et al., 2004) and oxytocin (Brailoiu et al., 2002), in the hypothalamic paraventricular and supraoptic nucleus. In addition, apelin colocalized densely with adrenocorticotrophin in corticotrophs and less densely with growth hormones in somatotropes in the anterior pituitary (Reaux-Le Goazigo et al., 2007). The authors also observed strong apelin receptor protein and mRNA expression in adrenocorticotrophin-positive and -negative cells of the pituitary, suggesting autocrine/paracrine actions of apelin-apelin receptor signaling in the pituitary.

In peripheral tissues, high levels of apelin have been reported in the mammary gland, heart, lung, and adipose tissue (Habata et al., 1999; Kawamata et al., 2001; Medhurst et al., 2003). Moderate to low levels were expressed in the kidney, adrenal gland, intestine, ovary, skeletal muscle, vas deferens, testes, and uterus (Lee et al., 2000; O’Carroll et al., 2000; Kawamata et al., 2001; Medhurst et al., 2003). In addition, Habata et al. (1999) reported in pregnant and lactating rats that the highest mRNA expression was during parturition, although compared with controls, transcript levels were increased during lactation. They also identified the peptide in bovine milk and colostrum, as well as human milk. In line with this, Mesmin et al. (2011) discovered ∼46 endogenous apelin peptides including apelin-13, apelin-17, apelin-22, and apelin-36 in milk and colostrum. Previously, apelin-13 and apelin-17 peptides have been found in rat hypothalamus (De Mota et al., 2004).

### C. Apelin Distribution in Mouse Tissues

So far, only a single real time quantitative polymerase chain reaction study investigated the distribution of apelin in mouse tissues, where the highest mRNA expression was reported in the brain, with moderate levels in the heart, kidney, and lungs while lower levels were found in the testes, uterus, muscle, spleen, and ovary (Medhurst et al., 2003).

### D. Elabela/Toddler Distribution in Human, Rat, and Mouse Tissues

Since ELA is a recent discovery, the distribution of *APELA* mRNA and the peptide in human and rodent tissues has not been thoroughly investigated yet. However, ELA mRNA has been shown to be developmentally regulated, with the inner cell mass of the blastocyst showing highest expression, and is downregulated upon differentiation (Ho et al., 2015). See section XII.A for further details of the role of ELA in embryonic development. Initial studies found that, alongside its expression during embryonic development, ELA is also expressed in adult human kidney and prostate tissues (Chng et al., 2013; Wang et al., 2015b). *APELA* transcripts have been reported in human blood vessels, with the highest levels detectable in arteries compared with veins and lower levels in human heart and lung tissue (Yang et al., 2017b).

In the rat, Deng et al. (2015) showed that ELA was exclusively expressed in the adult kidney compared with very low levels of apelin and apelin receptor. Recently, Perjés et al. (2016) demonstrated that although ELA mRNA was detectable in the adult rat heart (albeit at very low levels compared with apelin), this expression was mainly localized to noncardiomyocytes, especially endothelial cells and fibroblasts.

## VIII. Synthetic Agonists

Given the evidence that apelin treatment can be beneficial in a number of models of disease (section XI), it is unsurprising that there have been many attempts to produce synthetic apelin agonists with improved characteristics (Table 3). Such improvements have largely focused on increasing half-life due to the potential limitations of apelin therapeutically as a short-lived peptide. Many of these efforts have produced modified peptides based on the smallest active fragments of apelin, employing techniques such as polyethylene glycol (PEG-)ylation, the addition of unnatural amino acids, and cyclization. One anticipates that soon modified peptides based on the structure of the ELA will be identified and characterized. Finally, although small molecule synthetic agonists would prove to be most useful, only a few small molecule agonists have been reported, and these have generally proven unsuitable for experimental or therapeutic use.

**TABLE 3:** Some of the key agonists at the apelin receptor, their binding affinities, and whether they demonstrate bias compared with [Pyr^1^]apelin-13 (the predominant apelin isoform in the cardiovascular system (Maguire el al., 2009; Zhen et al., 2013) Endogenous agonists are denoted by “(E).”

Ligand	Action	Binding Affinity	Units	Bias	References
[Pyr^1^]Apelin-13 (E)	Full Agonist	7.0–8.8	pIC_50_	—	Kawamata et al., 2001
					Medhurst et al., 2003
Apelin-13 (E)	Full Agonist	8.8–9.5	pIC_50_	—	Fan et al., 2003
					Hosoya et al., 2000
					Medhurst et al., 2003
Apelin-17 (E)	Full Agonist	7.9–9.0	pIC_50_	*β*-arrestin	El Messari et al., 2004
					Medhurst et al., 2003
Apelin-36 (E)	Full Agonist	8.2–8.6	pIC_50_	—	Fan et al., 2003
					Hosoya et al., 2000
					Kawamata et al., 2001
					Medhurst et al., 2003
Elabela/Toddler-11 (E)	Full Agonist	7.2	pIC_50_		Yang et al., 2016
Elabela/Toddler-21 (E)	Full Agonist	8.7	pIC_50_	*β*-arrestin	Yang et al., 2016
Elabela/Toddler-32 (E)	Full Agonist	8.7	pIC_50_	*β*-arrestin	Yang et al., 2016
MM07	Full Agonist	9.5	pEC_50_	G protein	Brame et al., 2015
CMF-019	Full Agonist	8.6	pIC_50_	G protein	Read et al., 2016
ML233	Full Agonist	—	—	—	Khan et al., 2011
E339-3D6	Full Agonist	6.4	p*K*_i_	—	Iturrioz et al., 2010a

### A. Peptide Modifications and Discovery of Biased Ligands

PEGylation of certain drugs has previously been shown to improve their pharmaceutical properties and has led to 12 drugs in the clinic since 1990 (Turecek et al., 2016). For peptides, these benefits for the most part consist of an improvement in half-life through shielding from proteolytic enzymes. Attempts to PEGylate apelin have met with reasonable success. Jia et al. (2012) demonstrated that PEGylation of apelin-12 resulted in a 400-fold loss in binding affinity but that N-terminal PEGylation of apelin-36 with a 40-kDa PEG conjugate was tolerated. Moreover, in vivo assessment of ventricular ejection fraction following 20-minute infusion of PEGylated apelin-36 maintained potency. These inotropic effects could still be observed 100 minutes following cessation of infusion, whereas the response was already lost after 30 minutes using the endogenous peptide. In another study, Murza et al. (2012) illustrated that modification of C-terminal and central amino acids by addition of (PEG)_4_, (PEG)_6_, and (Ala)_4_ linkers to apelin-13 was able to extend plasma stability. These studies support PEGylation as a means to improve the plasma stability of apelin peptides without compromising functionality if the modifications are made appropriately.

Acylation, typically using fatty acids, has also provided significant extension of peptide half-life in vivo. The general strategy has been to increase the hydrodynamic radius coupled with increased plasma-protein binding to reduce metabolism and the rate of excretion via the kidney (Juhl et al., 2016; O’Harte et al., 2018a, see Section XI.E).

Low molecular weight molecules such as apelin are rapidly cleared via the kidneys, but conjugation to domain antibodies (the smallest stable fragment that can be created) that bind to serum albumin when injected into the plasma can potentially increase serum half-life to that of albumin itself (∼3 weeks). This method has been used to extend the plasma half-life of glucagon-like peptide-1 (Lin et al., 2015), where the peptide ligand-antibody conjugate was generated using fusion techniques but is limited to using the 20 genetically encoded amino acids. An alternative strategy has been to use a modified peptide MM202 [QRPRLSHKGP-Nle-P-(3,4,5 trifluoro)F], containing unnatural amino acids. This analog was designed to have high affinity for the apelin receptor and to be resistant to peptidase degradation (see below) and chemically linked to an anti-serum albumin domain antibody (AlbudAb) using maleimide chemistry. This was via a (PEG)_4_ linker at the pyroglutamate on the N terminus to extend half-life in blood. In competition binding experiments in human heart, MM202-AlbudAb bound with high affinity (*K*_i_ = 0.4 nM) to the apelin receptor, similar to the endogenous ligand [Pyr^1^]apelin-13 (*K*_i_ = 1.5 nM), retained potent agonist activity in *β*-arrestin and cyclic AMP assays and crucially the antibody retained the desired high binding affinity to human serum albumin (*K*_D_ = 0.95 nM). Agonist properties were retained in vivo. In Sprague-Dawley rats, MM202-AlbudAb at a concentration of 5 nmol, significantly reduced left ventricular systolic and arterial pressure and increased cardiac contractility and output (Read et al., 2019). These results demonstrate for the first time that chemically conjugating an apelin mimetic peptide to AlbudAb structure retains agonist activity and has wider applicability to other peptides modified by the addition of unnatural amino acids.

The addition of unnatural amino acids can help to improve stability and potency. For apelin-13 the key modifications have generally been made at the C terminus, as this is where cleavage by ACE2 occurs to remove phenylalanine. Wang et al. (2013b) reported two apelin analogs that were resistant to cleavage of this phenylalanine residue and identified that one was effective as an apelin mimetic, being able to protect against ischemia-reperfusion injury both in vivo and ex vivo. Similarly, Murza et al. (2015) focused on replacing the terminal phenylalanine with unnatural amino acids and identified several peptides with improved affinity. In particular, the use of large aromatic groups significantly improved binding, with F13Tyr(OBn) displaying a 60-fold improvement over the native peptide and, remarkably, the alpha methylated D-analog, F13(*α*Me)DTyr(OBn), displaying picomolar affinity. Interestingly, they reported that these molecules were more effective than apelin-13 in preventing cAMP accumulation but were equipotent in *β*-arrestin recruitment, suggesting that modifications at the C terminus of the apelin peptide may be important for imparting biased signaling properties. Recently, a group reported that through a combination of using unnatural amino acids and lipidation of apelin-13, they were able to produce an apelin agonist with a greatly enhanced in vitro half-life of 29 hours in rat plasma (Juhl et al., 2016). Such a finding is remarkable, particularly since they report that the molecule maintains a high potency at the receptor.

Cyclization of peptides results in conformational restriction and can improve stability by preventing peptidase action, especially in small peptides such as apelin, where there is ordinarily a large amount of free movement. Initial experiments showed that cyclized apelin-12 peptides could retain potency in recombinant human apelin receptor expressing cell lines (Hamada et al., 2008). Experiments by Macaluso and Glen (2010), as discussed earlier, demonstrated the importance of the RPRL motif and some following attempts focused on cyclization around the RPRL motif to extend plasma half-life. From this approach, MM07 was identified by Brame et al. (2015) and showed greater stability over [Pyr^1^]apelin-13, with a half-life of 17 minutes compared with 2 minutes in rat plasma.

A further limitation of endogenous apelin such as [Pyr^1^]apelin-13 is that, in addition to activating G proteins, signaling involves coupling to *β*-arrestin (Evans et al., 2001), resulting in receptor desensitization (Masri et al., 2006, and has be characterized in detail by Pope et al. (2016). This reduces the efficacy of repeated dosing by apelin ligands. MM07 was found to display G protein bias at the apelin receptor, with reduced potency at *β*-arrestin-mediated desensitization in cell and organ bath assays. Importantly, in the cell-based functional assay (DiscoverRx PathHunter; Eurofins DiscoverX Corporation, Fremont, CA) used to measure *β*-arrestin recruitment, the full-length apelin receptor is fused at the C terminus with a fragment of *β*-galactosidase enzyme and is otherwise unmodified. The complementary fragment of the enzyme is linked to *β*-arrestin. Ligand binding induces *β*-arrestin recruitment, forcing complementation of the two *β*-galactosidase enzyme fragments. The resulting functional enzyme hydrolyzes substrate to generate a chemiluminescent signal directly proportional to ligand activity (Bassoni et al., 2012).

This G protein bias was maintained in human volunteers, producing the expected vasodilatation when tested using both Aellig hand-vein and forearm venous occlusion plethysmography techniques. As predicted, repeated administration of MM07 to the human forearm vasculature did not result in a reduction of the response. Apelin peptide (but not the target receptor) is significantly reduced in cardiovascular disease such as heart failure and pulmonary arterial hypertension (PAH) (Goetze et al., 2006; Alastalo et al., 2011; Chandra et al., 2011; Yang et al., 2017b), where the therapeutic hypothesis is that apelin receptor agonists are needed to replace the missing endogenous peptide. Desensitization and the development of tolerance are a limiting factor for many agonists targeting GPCRs.

Importantly, as proof of concept, MM07 significantly reduced the elevation of right ventricular systolic pressure and hypertrophy in the monocrotaline rat model of PAH. Monocrotaline-induced changes in cardiac structure and function, including right ventricular end-systolic and end-diastolic volumes, ejection fraction, and left ventricular end-diastolic volume, were attenuated by MM07 (Yang et al., 2019). MM07 also significantly reduced monocrotaline-induced muscularization of small pulmonary blood vessels. MM07 stimulated endothelial nitric oxide synthase phosphorylation and expression, promoted proliferation, and attenuated apoptosis of human pulmonary arterial endothelial cells in vitro. These findings suggest that chronic treatment with MM07 is beneficial in this animal model by addressing disease etiology. These data support the development of G protein-biased apelin receptor agonists with improved pharmacokinetic profiles for use in human disease.

Murza et al. (2017) also reported on biased cyclized apelin-like peptides. Instead of cyclizing around the RPRL motif, they replaced His^7^ and Met^10^ with allylglycine and cyclized between these residues. They further replaced the C-terminal phenylalanine residue with Tyr(OBn) and produced various modifications upon this scaffold structure. They were able to display that replacement of the Tyr(OBn) residue with nonaromatic residues or transfer into the ring structure markedly reduced the ability to recruit *β*-arrestins. Such bias toward G protein signaling was similar to MM07. However, unlike MM07, which produced clear decreases in blood pressure in human volunteers, Murza et al. (2017) reported that their G protein biased compounds had a reduced ability to induce hypotensive effects in rats. Such an observation is supported by the report of modified apelin-17 peptide fragments biased toward the *β*-arrestin pathway, which were more able to induce decreases in blood pressure in rats (El Messari et al., 2004). Of the compounds described by Murza et al. (2017), compound 18 is notable, despite its low micromolar affinity. This is due to its lack of the RPRL motif, a motif previously thought critical to binding. Additional studies that have looked at cyclization away from the RPRL motif include a patent by Novartis (Basel, Switzerland) (Golosov et al., 2013) producing peptides based on [Pyr^1^]apelin-13 with a range of bridging linkers, including esters, disulfides, amides, and short polyether bridges. Of these, some showed improved half-lives and a few were reported to possess similar potency to [Pyr^1^]apelin-13. McAnally et al. (2017) also identified biased agonists displaying preferential signaling via either G protein or *β*-arrestin by screening ∼450 compounds designed rationally but also with a random side-chain substitution approach. Two of the most promising compounds that were either G protein or *β*-arrestin biased were selected to be characterized further, including extracellular signal regulated kinases 1/2 activation assays and radioligand binding.

Two apelin-36 analogs, designated N-58 (where Lys^28^ was substituted by Ala^28^) and N-140 (a PEGylated analog of N-58, to increase plasma half-life), were reported to induce metabolic effects in vivo, but in contrast to the native peptide, apelin-36, did not lower blood pressure, suggesting actions that might be independent of canonical apelin receptor signaling (Galon-Tilleman et al., 2017). In a subsequent study, both N-58 and N-140 competed for the binding of [^125^I]-apelin in human heart homogenates with the expected nanomolar affinities. N-58 and N-140 were 100- and 2000-fold, respectively, less potent at recruiting *β*-arrestin in cell-based assays compared with the apelin-36 but inhibited forskolin-induced cAMP release, a measure of G_*α*i_ pathway activation, with nanomolar potencies. These results support the hypothesis that the modifications may bias the response to N-58 and N-140 toward G_*α*i_ or G_*α*q_ pathways that were previously shown to be important in mediating the metabolic effects of apelin peptides (Nyimanu et al., 2018).

While many studies have focused on unnatural amino acid addition, cyclization, and PEGylation as a means to improve the pharmacological properties of peptides, one very interesting study has looked at the use of pepducins. These are lipidated peptide sequences usually 8–16 amino acids in length and based on the structure of the intracellular loops of G protein-coupled receptors. McKeown et al. (2014) produced a complete array of N-lipidated 12-mer peptide sequences for the apelin receptor and 7 demonstrated agonism at 1 or 10 *µ*M and were resynthesized. One compound (designated compound 1), displayed activity and was found to be selective for the apelin receptor. This result was not unexpected given the sequence from which it was based is a poorly conserved region of the GPCR. Furthermore, to confirm selectivity they produced the peptide with D-amino acids as well as without the N-terminal palmitate lipidation and demonstrated a complete loss of activity. Such a unique approach is very interesting and could widely be applicable to drug discovery at orphan GPCRs.

### B. Small Molecules and Discovery of Biased Ligands

One of the first reports of a peptidomimetic small molecule was E339-3D6 (Iturrioz et al., 2010a, Table 3), which showed a reasonable affinity for the apelin receptor (*K*_i_ ∼400 nM in radioligand binding experiments) and selectivity over other related GPCRs. However, with a molecular weight of 1400 Da, the compound would not qualify as a small molecule. Moreover, it was later shown to be a mixture of polymethylated species (Margathe et al., 2014). The constituents of this mixture were separated and some were found to bind to the apelin receptor with improved affinity compared with the parent mixture, although these affinities were still relatively low and the molecules had comparatively high molecular weights for compounds to be classed as drug-like.

Another attempt to identify small molecule agonists used a high-throughput screen of ∼330,600 molecules and found ML233 (Khan et al., 2011, Table 3). This molecule conformed to the traditional definition of a small molecule with a molecular weight of 359 Da, under the usual 500 Da cut-off defined by Lipinski’s rules. Unfortunately, it showed poor solubility in saline at room temperature, and its structure indicated it would likely be toxic through being both a Michael acceptor and possessing a reactive activated quinone group (Lagorce et al., 2015).

Recently, several studies and patents have reported the development of more suitable drug-like small molecules. Narayanan et al., (2016) used a drug library of approximately 100 compounds screened in a high-throughput Ca^2+^ mobilization assay and identified four compounds based on the same structural scaffold that they designated compound 1. By experimenting with three key side chain sites, they explored the changes that were tolerated by the scaffold and suggested that this could act as a starting point for the production of more suitable drug-like small molecules. Although these molecules were small (molecular weight ∼500 Da), they possessed relatively low potencies in the micromolar range. The important question will be whether potency can be improved by side-chain experimentation or if it is a limit of the scaffold itself.

Further studies explored the properties of a molecule designated CMF-019 (Read et al., 2016, Table 3) derived from the patent (Hachtel et al., 2014). This molecule had a suitably low molecular weight (455 Da) and high nanomolar affinity for the apelin receptor. The high binding affinity translated into a functional effect with similar levels of cAMP inhibition to [Pyr^1^]apelin-13 observed in cells expressing the recombinant human apelin receptor. It was also able to promote cardiac contractility (Read et al., 2016) and vasodilatation (Read et al., 2017, 2019) in vivo, as one would expect for an apelin agonist. Interestingly, the molecule showed much lower activity in recruiting *β*-arrestin and in internalizing the apelin receptor, suggesting that it was highly biased toward the G protein signaling pathways. Such bias could be very useful as an apelin therapy by enabling it to activate the beneficial G protein pathways without internalizing the receptor and thereby maintaining the response over time. Importantly, although the bias of the compound was identified in vitro (Read et al., 2016), it has since been shown to translate to reduced receptor internalization in vivo (Read et al., 2017). A potential drawback of this molecule was a comparatively high logP, which resulted in limited solubility and prevented high doses from being administered in the in vivo studies. Nevertheless, it is arguable that CMF-019 provides the most suitable scaffold upon which to explore new small molecule apelin agonists, as it already possesses high affinity to the apelin receptor and novel syntheses have already been explored (Trifonov et al., 2018).

**Fig. 7.**
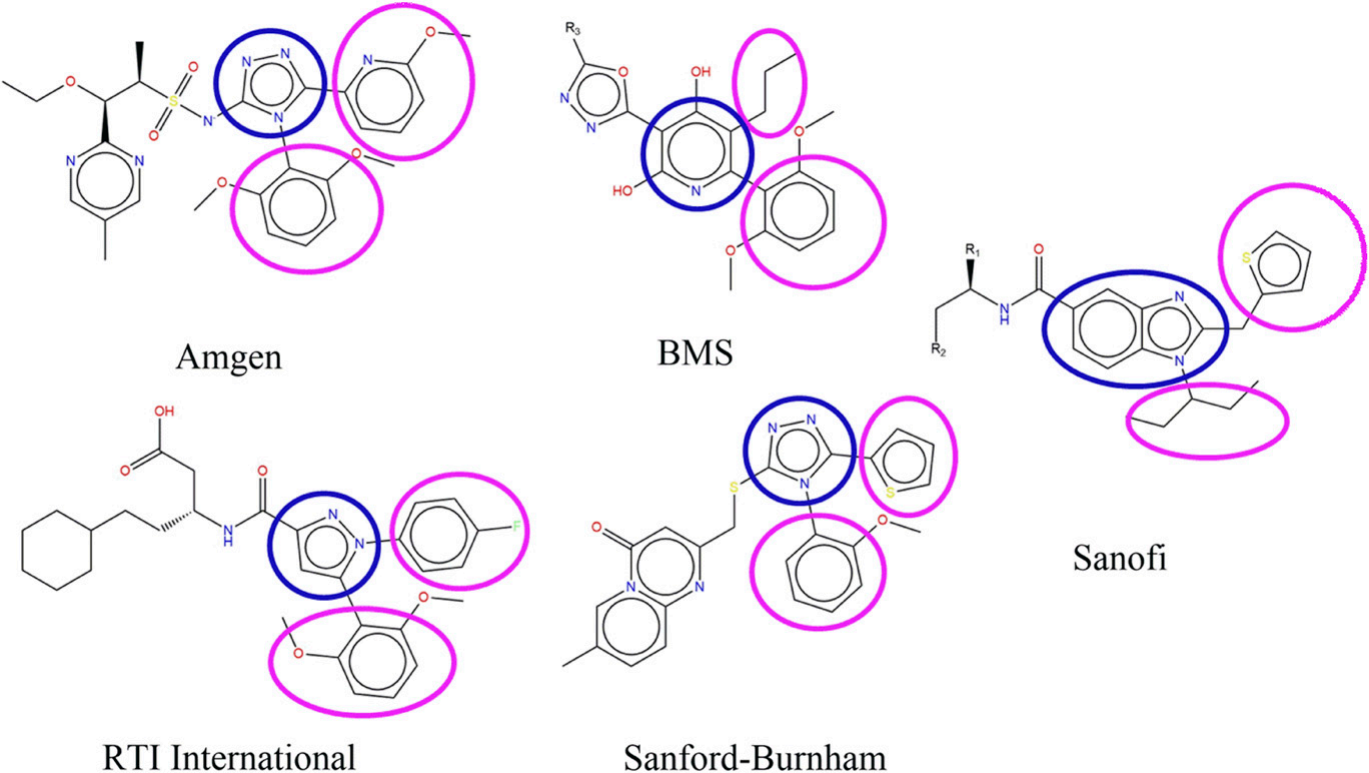
The scaffold structures of five reported series of small molecule apelin agonists from Amgen (Chen et al., 2017), Bristol-Myers Squibb (Myers et al., 2017), RTI International (Narayanan et al., 2016), Sanford-Burnham (Pinkerton and Smith, 2015), and Sanofi (Hachtel et al., 2014). CMF-019 is derived from the Sanofi series. All of these molecules possess a broadly similar structure, consisting of two hydrophobic groups (circled in pink) extending from a heterocyclic core group (in blue).

**Fig. 8.**
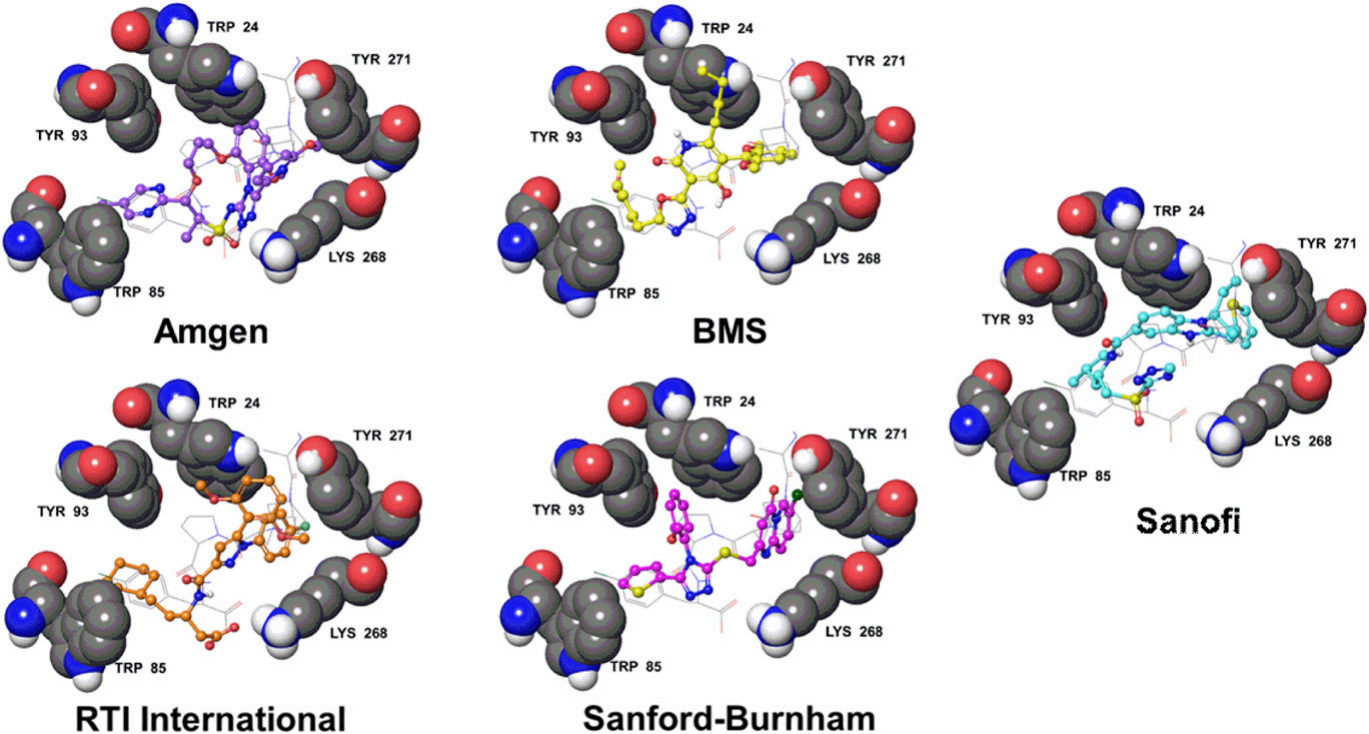
The apelin receptor structure from PDBID 5VBL (Ma et al., 2017) with docked poses of five series of apelin agonists. The apelin receptor residues, W^24^, W^85^, Y^93^, K^268^, and Y^271^ are labeled and displayed as gray space filling. The structure of the four C-terminal residues of the apelin analog from PDBID 5VBL are displayed as gray sticks. The receptor and peptide are overlaid with docked poses of an Amgen (violet balls and sticks), a Bristol-Myers Squibb (yellow balls and sticks), an RTI International (orange balls and sticks), a Sanford-Burnham (magenta balls and sticks), and a Sanofi (cyan balls and sticks) small molecule apelin agonist. Only polar hydrogens are shown. Structures were derived from the following patents: Amgen (Chen et al., 2017), Bristol-Myers Squibb (Myers et al., 2017), RTI International (Narayanan et al., 2016), Sanford-Burnham (Pinkerton and Smith, 2015), and Sanofi (Hachtel et al., 2014). Pinkerton and Smith (2015) confirmed the Sanford-Burnham apelin compounds were selective vs. the angiotensin II receptor (AT_1_), the most closely related GPCR, with no significant off target binding.

Several series of small molecule apelin agonists that share some structural similarity to CMF-019 were recently reported by Amgen (Chen et al., 2017), Bristol-Myers Squibb (Myers et al., 2017), RTI International (Narayanan et al., 2016), and Sanford-Burnham (Pinkerton and Smith, 2015). They all possess two hydrophobic substituents extending from a heterocyclic core, reminiscent of the Sanofi series of compounds from which CMF-019 is derived (Hachtel et al., 2014; Fig. 7). The structural similarities between these compounds suggest that they may bind within the same site in the apelin receptor and computational docking experiments support this (Fig. 8). However, it is also possible that the compounds bind to an allosteric site distinct from the apelin binding site and modulate activity through an alternative allosteric mechanism. The identification of similar patterns of substituent structure activity relationships may further support the hypothesis of a common binding site. It will be fascinating to see if other compound series display G protein bias similar to CMF-019. To our knowledge, these experiments have not yet been performed and reported. The discovery that the apelin receptor is tractable to biased agonism with CMF-019 suggests this will become an area of expanding interest.

### C. Summary

There have been significant advances in the development of synthetic agonists at the apelin receptor. Studies utilizing peptide modification have been able to improve plasma stability and affinity through a variety of methods, including PEGylation, acylation, cyclization, and the addition of unnatural amino acids. Initial studies to identify small molecules have had significant issues. However, recently more suitable small molecules have emerged, in particular CMF-019. These will be useful in future studies as tool compounds and in the development of potential small molecule therapeutics.

## IX. Synthetic Antagonists

Just as there has been much interest in the development of agonists at the apelin receptor, antagonists at the receptor have also been sought (Table 4). So far, although a number have been described, there is still a need to find more suitable molecules for use in both in vitro and in vivo studies. Apelin antagonists might also possess therapeutic potential themselves and recently there has been interest in their use for the treatment of cancer due to their anti-angiogenic effects.

**TABLE 4:** Some of the key antagonists at the apelin receptor and their binding affinities MM54, an antagonist at the *β*-arrestin and internalization pathway, consists of a cyclized peptide based around the RPRL motif. MM54 has been tested for selectivity (Bowes et al., 2012) against over 50 GPCRs (including the most closely related angiotensin II receptor AT_1_) and ion channels. ALX40-4C and protamine both consist of a series of positively charged amino acids and display low binding affinities and likely low selectivity for the apelin receptor.

Ligand	Action	Binding Affinity	Units	References
MM54	Antagonist	8.2	p*K*_i_	Macaluso et al., 2011
ALX40-4C	Antagonist	5.5	pIC_50_	Zhou et al., 2003b
ML221	Antagonist	—	—	Maloney et al., 2012
Protamine	Antagonist	6.4	p*K*_i_	Le Gonidec et al., 2017

One of the first antagonists described was F13A, an apelin analog in which the C-terminal phenylalanine was mutated to an alanine residue. Initially, De Mota et al. (2000) described alterations in the apelin peptide K17F [a 17-amino acid chain stretching from lysine (1) to phenylalanine (17) of the apelin sequence] and identified that the first four amino acids were not required for activity, thus describing apelin-13. They further suggested that substitution of the terminal phenylalanine for alanine completely abolished activity. Lee et al. (2005) later described the antagonist activity of F13A in spontaneously hypertensive rats through the blockade of hypotensive responses induced by apelin. Despite these two studies suggesting F13A does not possess agonist activity and the latter describing it as an antagonist, other studies dispute this. Both Fan et al. (2003) and Medhurst et al. (2003) describe it in alanine mutagenesis studies as an agonist as discussed in section V. Fan et al. (2003) reported that F13A possessed equal potency to apelin-13 in inducing receptor internalization, meanwhile Medhurst et al. (2003) reported a decrease in both binding affinity to the apelin receptor (2- to 14-fold) and in potency in a FLIPR assay (8-fold). In a recent study, Yang et al. (2017a) also reported on the activity of F13A, demonstrating that it possessed similar activity to [Pyr^1^]apelin-13_(1–12)_ in an in vitro *β*-arrestin recruitment assay and that it was also able to contract saphenous vein ex vivo with subnanomolar potency. These studies suggest, therefore, that F13A retains agonistic activity and is not in fact an antagonist. Perhaps the best explanation is that it acts as a partial agonist; this would explain its ability to antagonize hypotensive responses to apelin (Lee et al., 2005) and its agonist activity when tested on its own (Medhurst et al., 2003; Yang et al., 2017a).

Another reported peptide antagonist is ALX40-4C (Zhou et al., 2003b, Table 4). It was originally identified at the CXCR4 chemokine receptor in the context of HIV infection [it was through this that Zhou and colleagues identified its activity at the apelin receptor that acts as a coreceptor for HIV infection (Section XI.G)]. They demonstrated direct binding to the receptor with an IC_50_ of 2.9 *µ*M and inhibition of internalization induced by apelin. Despite antagonistic effects, there is little evidence that it could be used as a selective apelin antagonist. Indeed, it was identified initially as a CXCR4 antagonist and, although its sequence of a string of highly charged amino acids might make it effectual in blocking the apelin receptor, it is unlikely to aid selectivity over other GPCRs. Without selectivity it is unlikely to find use either as a pharmacological tool compound or in therapy.

A peptide ligand, MM54 (Table 4), designed based on known functionality of apelin sequences at the apelin receptor would be expected to show more promising selectivity (Macaluso et al., 2011). Macaluso and colleagues started with the RPRL motif to produce two cyclic “anchors” with a dipeptide linker in between. This was based on the message-address hypothesis of peptide agonists. In this case, RPRL is the address, while HKGPMPF is the message. The inclusion of two addresses and a scrambled message was hypothesized to produce antagonistic effects. They hypothesized that the linker could be altered to act as a “switch,” thus alternating receptor stabilization between agonist and antagonist states. MM54, bound to the human apelin receptor in left ventricular (LV) heart homogenates with submicromolar affinity (*K*_D_ = 320 nM). MM54 antagonized apelin mediated *β*-arrestin recruitment (*K*_B_ = 118 nM) and receptor internalization (*K*_B_ = 1.3 *µ*M) but, interestingly, activated the G protein pathway as an agonist (EC_50_ = 1.4 *µ*M), suggesting pathway bias (Davenport et al., 2018). This was confirmed in vivo in humans, where at the highest concentration tested (100 nmol/min infusion rate), MM54 caused a significant absolute increase in forearm blood flow compared with control arm, representing a ∼75% change from baseline. In the hand vein, MM54 caused a significant concentration-dependent dilatation in veins over the concentration range tested, with the highest dose causing ∼60% reversal (Davenport et al., 2018). Recently Harford-Wright et al. (2017) reported on the use of MM54 to treat glioblastoma. In this study, they tested MM54 for selectivity for the apelin receptor by screening it in radioligand binding assays against a panel of 55 other GPCRs or ion channels.

A small molecule, ML221 (Table 4), displayed antagonistic action in the micromolar range to both cAMP and *β*-arrestin signaling through the apelin receptor (Maloney et al., 2012). However, it showed a poor pharmacokinetic profile with very limited solubility in aqueous media at pH 7.4 and poor stability and therefore is not recommended for in vivo studies. However, ML221 effectively antagonizes the action of apelin and ELA in vitro (Yang et al., 2017b) and is, therefore, useful for in vitro studies especially as it has good selectivity for the apelin receptor.

Most recently, the U.S Food and Drug Administration-approved compound, protamine (Table 4) was identified as an antagonist at the apelin receptor (Le Gonidec et al., 2017). This compound is usually used after cardiac surgery, where it binds to heparin to reverse anticlotting activity. The study demonstrates a binding affinity of 390 nM at the apelin receptor and full antagonistic activities against both G protein and *β*-arrestin signaling, as well as against in vivo dilatation in mice. Screening against several other homologous GPCRs, such as the AT_1_ receptor, suggests some degree of selectivity. However, caution is advised as the structure of protamine is based on a series of highly positively charged arginine residues and is reminiscent of ALX40-4C.

There is still a need for a selective antagonist at the apelin receptor, because currently available molecules possess limitations. Future studies will lead to selective, high affinity antagonists for use in pharmacological study as well as in therapy.

## X. Radiolabeled Ligands

Several radiolabeled ligands based on the structure of apelin have been designed against the apelin receptor and have mostly focused on apelin-13 or its pyroglutamated analog (Table 5). Fan et al. (2003) directly labeled apelin-13 with ^125^iodine producing [^125^I]apelin-13, which bound to the apelin receptor with a binding affinity of 0.63 nM. Katugampola et al. (2001), on the other hand, used [Pyr^1^]apelin-13 and found that [^125^I]-[Pyr^1^]apelin-13 bound to the receptor with an affinity of 0.35 and 0.33 nM in human left ventricle and right atria, respectively. Moreover, they demonstrated binding with a Hill slope close to unity, consistent with the identification of only one apelin receptor subtype in humans. Finally, this study looked at the kinetics of [^125^I]-[Pyr^1^]apelin-13 binding and demonstrated rapid association at 23°C with a half-time of 6 minutes followed by a slower dissociation with a half-time of 53 minutes. Other radiolabeling studies have used modified peptides to obtain suitable ligands. Hosoya et al. (2000) substituted Phe^77^ to a tyrosine residue to allow the addition of ^125^I using a lactoperoxidase. They then further substituted Met^75^ for a norleucine residue to prevent oxidation during the labeling process. The final ligand, [^125^I]-[Pyr^1^]-[Nle^75^, Tyr^77^]apelin-13, bound to the apelin receptor with an affinity of 20 pM and is commercially available. Similar modifications have been made for apelin-36 to produce [^125^I]-[Nle^75^, Tyr^77^]apelin-36, which binds to the apelin receptor with an affinity of 6.3 pM. For similar reasons to Hosoya and colleagues, Medhurst et al. (2003) oxidized Met^75^ when producing a tritiated [Pyr^1^]apelin-13 ligand. They found that the unoxidized ligand was unstable, while the Met^75^ residue was prone to spontaneous oxidation during tritiation, purification and storage. The final ligand [^3^H]-[Pyr^1^]-[Met^75^(*O*)]apelin-13 bound with an affinity of 2.5 nM to the apelin receptor.

**TABLE 5:** Some of the key radiolabeled ligands at the apelin receptor and their binding affinities

Ligand	Action	Binding Affinity	Units	References
[^125^I][Nle^75^,Tyr^77^]apelin-36 (human)	Full Agonist	11.2	p*K*_d_	Kawamata et al., 2001
[^125^I][Glp^65^, Nle^75^, Tyr^77^]apelin-13	Full Agonist	10.7	p*K*_d_	Hosoya et al., 2000
[^125^I][Pyr^1^]apelin-13	Full Agonist	9.5	p*K*_d_	Katugampola et al., 2001
[^3^H][Pyr^1^][Met(0)11]-apelin-13	Full Agonist	8.6	p*K*_d_	Medhurst et al., 2003
[^125^I]apelin-13	Full Agonist	9.2	p*K*_d_	Fan et al., 2003

## XI. Apelin Physiology and Pathophysiology

### A. Developmental Roles

ELA appears to be the principal developmental peptide at the apelin receptor. It was in part due to discrepancies in apelin receptor and apelin peptide knock-out zebrafish, as well as the fact that apelin is not expressed until much later in development than its receptor, that prompted the discovery of ELA (Pauli et al., 2014). Nevertheless, apelin has been shown to have some developmental roles. Kidoya et al. (2015) found that apelin, through its receptor, was important for arterial-venous alignment in the skin. They demonstrated that knockout mice of both the receptor and peptide were less able to deal with hot or cold heat stress, suggesting an important role in thermoregulation.

### B. Cancer

Apelin expression is elevated in a number of cancers, such as lung non-small cell carcinomas, gastroesophageal, glioblastoma, colon, hepatocellular, prostate, endometrial, and oral squamous cell carcinoma. It induces endothelial cell migration and proliferation, supporting a role in tumor neoangiogenesis; for a detailed review on the subject see Yang et al. (2016). The high expression of apelin in cancerous tissues raises the possibility of using it as a biomarker. Clinical trials are underway to determine if a reduction in serum apelin levels can be used to assess the efficacy of bevacizumab treatment as a measure of tumor vasculature normalization following similar observations in a mouse model (Zhang et al., 2016). In glioblastoma patients, high ELA expression has also been associated with poor survival, implicating the peptide as a potential biomarker for the disease (Ganguly et al., 2019). Furthermore, this correlation may be causative and it has been hypothesized that ELA has an oncogenic role promoting tumor progression. This has also been demonstrated in both in vitro and in vivo with ovarian clear cell carcinoma studies, where ELA was shown to contribute to cancer growth, progression, and migration, and knockout of ELA reduced the extent to which these processes occurred (Yi et al., 2017).

Directly targeting the apelin receptor with antagonists could prove a useful therapy if used in appropriate combination with current market drugs. In a recent study, Harford-Wright et al. (2017) reported on MM54 treatment of glioblastoma. They demonstrated that glioblastoma stem-like cells expressing higher apelin levels were better able to initiate tumor development. Furthermore, knocking down apelin with shRNA could reduce the number of progressing tumors in an in vivo implant model. They went on to look at pharmacological inhibition with MM54 and showed that it impaired the expansion of glioblastoma stem-like cells that were resistant to standard therapy in vitro. This translated to the in vivo tumor xenograft model with MM54 reducing tumor growth and promoting survival of transplanted mice. Interestingly, targeting the apelin receptor in this model proved beneficial by reducing vascularization and proliferation in the tumor and reduction in gliomagenesis via GSK3*β* signaling. However, another recent study has identified loss-of-function mutations in the apelin receptor in patients refractory to immunotherapy (Patel et al., 2017). They showed a role for the apelin receptor in modifying T-cell responses through the JAK-STAT pathway, leading to an augmentation of the interferon-*γ* response. This response appeared to be independent of canonical G protein signaling through the apelin receptor, and whereas the authors demonstrated that knocking down the apelin receptor in mouse melanoma models reduced the efficacy of T-cell-based therapies, they did not show whether an antagonist would do the same. This question should be addressed because, if the JAK-STAT response is ligand independent, this could avoid the potential complication of apelin receptor blockade, reducing host immune responses in cancer.

### C. Fibrosis

Apelin is involved in fibrotic mechanisms in a number of tissues, including kidney, liver, heart, and lung; for a detailed review, see Huang et al. (2016b). Intriguingly, apelin displays conflicting roles in these different tissues, protecting against renal, myocardial, and pulmonary fibrosis, while potentially promoting liver fibrosis.

In the kidney, plasma apelin levels are significantly lower in patients with autosomal dominant polycystic kidney disease compared with controls, and apelin levels correlated with estimated glomerular filtration rate (Kocer et al., 2016). The mechanism by which apelin is protective has been suggested to involve transforming growth factor *β* (TGF*β*) signaling with demonstrations that apelin is able to reduce renal interstitial fibrosis through suppressing tubular epithelial to mesenchymal transition via a Smad-dependent mechanism (Wang et al., 2014). Moreover, in a renal ischemia/reperfusion model in rats, apelin was downregulated and infusion of apelin-13 could reduce acute injury through suppression of TGF- *β*1 (Chen et al., 2015).

In addition to its involvement in renal fibrosis, TGF*β* is known to have roles in numerous fibrotic mechanisms (Leask and Abraham, 2004), including in the myocardium (Koitabashi et al., 2011). A downstream miRNA of TGF- *β*, miR-125b, has been shown to be upregulated in cardiac fibrosis and to inhibit apelin (Nagpal et al., 2016). This supports a role for apelin downregulation in fibrosis and provides a mechanism through which it may occur. It also suggests that apelin replacement could be beneficial. Indeed, it has been shown that infusion of various apelin isoforms is beneficial in several models of myocardial damage; for example, in mice with ligated coronary arteries to induce infarction and subsequent cardiac hypertrophy and fibrosis, apelin-13 was protective (Li et al., 2012). Furthermore, apelin recruited bone marrow cells and adenovirally mediated overexpression of apelin in these cells enhanced cardiac repair (Li et al., 2013). In isolated perfused rat hearts, infusion of apelin-12 analogs were beneficial in reducing myocardial infarct size, cell membrane damage, and cardiac dysfunction. This protection was associated with activation of various cellular kinases, leading to activation of numerous downstream targets, including nitric oxide synthase, mitochondrial ATP-sensitive potassium channel channels, and the sarcolemmal Na^+^/H^+^ and Na^+^/Ca^2+^ exchangers (Pisarenko et al., 2015a). Additionally, these apelin-12 analogs reduced reactive oxygen species, resulting in a reduction in cellular membrane damage (Pisarenko et al., 2015b). Other reports have suggested that apelin-13 can protect against in vivo myocardial ischemia-reperfusion by inactivating glycogen synthase kinase 3*β*, preventing the opening of the mitochondrial permeability transition pore involved in the reperfusion injury salvage kinase pathway (Yang et al., 2015b). In ApoE-knockout mice infused with angiotensin II to induce atherosclerosis of the coronary arteries, coinfusion of apelin-13 could activate nitric oxide and thereby attenuate atherosclerosis of these vessels, as well as prevent vascular remodeling in a vein graft model (Chun et al., 2008).

Pulmonary fibrosis occurs in many lung pathologies and there is evidence that apelin can help to prevent this. In a rat model of bronchopulmonary dysplasia (a chronic disease that normally occurs in premature infants treated with oxygen and positive pulmonary pressure), treatment with apelin-13 reduced pulmonary fibrin deposition and inflammation (Visser et al., 2010). Meanwhile, in a rat model of acute respiratory distress syndrome induced by oleic acid, apelin-13 treatment could reduce inflammation and lung injury. F13A also reduced oleic acid induced histopathological changes (Fan et al., 2015a).

Unlike in kidney, myocardial, and lung fibrosis where apelin is beneficial, in hepatic fibrosis the role of apelin is less clear. In cirrhotic rat liver, both the apelin receptor and peptide are increased, meanwhile in patients with cirrhosis, circulating apelin levels are increased (Principe et al., 2008). However, treatment of cirrhotic rats with F13A led to an alleviation of liver fibrosis (Principe et al., 2008; Reichenbach et al., 2012). This might suggest that the lower activity of F13A is sufficient to partially antagonize the elevated endogenous apelin, supporting a role as a partial agonist. Alternatively, it is possible that the elevation of apelin is a protective mechanism, and hence, F13A is protective as an agonist. Further studies are required to clarify the role of apelin in liver fibrosis.

Overall, apelin is beneficial in a number of different disease models of tissue fibrosis in kidney, heart, and lung tissues; however, it may accentuate liver fibrosis. Such a role in myocardial and pulmonary fibrosis could be particularly useful in treating heart failure and PAH where substantial fibrosis is known to occur in both organs (Murtha et al., 2017; Thenappan et al., 2018) and where apelin has already been identified as a potential therapeutic strategy.

### D. Pulmonary Arterial Hypertension and Heart Failure

#### 1. Preclinical Studies and Plasma Levels in Patients with Pulmonary Arterial Hypertension

The vasodilatory and inotropic effects of apelin signaling have made it of particular interest as a potential therapeutic for diseases such as PAH and heart failure (HF) (Yang et al., 2015a; Kuba at al., 2019). Interestingly, there is significant evidence that the apelin peptide is downregulated in both diseases. This has been found in plasma from patients in Group 1 PAH (including idiopathic PAH, PAH associated with drugs or toxins, and autoimmune disease, Goetze et al., 2006; Alastalo et al., 2011; Chandra et al., 2011; Yang et al., 2017b). The main source of apelin and ELA in the human vasculature is from the endothelium. Apelin is localized to the small secretory vesicles of the constitutive rather than the regulated pathway (Yang et al., 2017b). Following continuous release from the constitutive pathway, the peptides are thought to act in an autocrine or paracrine manner, predominantly on apelin receptors present on endothelial cells to release vasodilators. Plasma levels of ELA (0.34 nmol/l) and apelin (0.26 nmol/l) measured in the same healthy volunteers (Yang et al., 2017b) are comparatively low and may represent overspill, in agreement with locally released rather than circulating mediators. Apelin isoforms have comparable potencies at the apelin receptors expressed on cardiomyocytes and vascular endothelial and smooth muscle cells from human cardiovascular tissue isolated in organ baths (Table 6). Plasma levels are comparable to EC_50_ values measured in isolated human tissue assays and are likely to be sufficient to have functional effects.

**TABLE 6:** Apelin isoforms have comparable potencies at the apelin receptor expressed on cardiomyocytes, vascular endothelial and smooth muscle cells from human isolated cardiovascular tissue EC_50_ values are geometric means. Values are mean ± S.E.M.

Action	[Pyr^1^]Apelin-13			Apelin-13			Apelin-36		
	EC_50_	pD_2_	E_MAX_	EC_50_	pD_2_	E_MAX_	EC_50_	pD_2_	E_MAX_
Inotropy*^a^*	0.1	9.9 ± 0.2	49%	0.08	10.1 ± 0.3	64%	0.04	10.4 ± 0.2	39%
Vasodilatation*^b^*	1.6	8.8 ± 0.1	39%	0.6	9.2 ± 0.2	51%	0.8	9.1 ± 0.2	43%
Vasoconstriction*^c^*	1.6	8.8 ± 0.5	30%	0.8	9.1 ± 0.2	19%	0.6	9.2 ± 0.5	17%

EC_50_, the concentration (nmol/l) of apelin peptide producing 50% of the maximum response to that peptide; E_MAX_, maximum response, expressed as a % of a reference stimulus; pD_2_, negative log_10_ EC_50_.

^*a*^ Human, electrically paced atrial strip. Maximum response is % inotropic response to calcium (8.95 mmol/l).

^*b*^ Endothelium-dependent vasodilatation in human mammary artery pre-constricted with ET-1. Maximum response (E_MAX_) is % reversal of ET-1 response.

^*c*^ Contraction of endothelium-denuded saphenous vein. Maximum response (E_MAX_) is %KCL (100 mmol/l). Tissues were maintained in organ baths at 37°C in oxygenated physiologic saline (Maguire et al., 2009).

In agreement with lower plasma levels, pulmonary artery endothelial cells (Kim et al., 2013) and pulmonary microvascular endothelial cells (Alastalo et al., 2011) have lower levels of apelin when cultured from PAH patients compared with cells from control donors. Mice where the gene encoding apelin has been deleted, develop more severe pulmonary hypertension under hypoxia (Chandra et al., 2011). They also display higher right ventricular systolic pressure, increased muscularization of the alveolar wall arteries, and greater loss of pulmonary microvasculature in response to hypoxia compared with wild-type controls (Chandra et al., 2011). In all animal models of PAH studies to date, apelin is reduced in the right ventricle in the monocrotaline rat (Falcão-Pires et al., 2009) and Sugen 5416 with hypoxia-induced (Drake et al., 2011; Frump et al., 2015) rat models of PAH. Apelin levels are correlated with contractile and diastolic function of the right ventricle in the latter model (Neto-Neves et al., 2017). Despite downregulation of apelin, the receptor is still present in PAH tissue (Andersen et al., 2009; Kim et al., 2013). Daily injection of apelin (Falcão-Pires et al., 2009; Alastalo et al., 2011), agents that stimulate apelin expression (Spiekerkoetter et al., 2013; Bertero et al., 2014; Nickel et al., 2015), or downstream mediators of the apelin pathway (Kim et al., 2013) attenuate the development of PAH in these models. As previously discussed (section V.B), ELA is also downregulated in humans as well as MCT and Sugen/hypoxia models. Like apelin, daily injection of ELA is able to replace the missing endogenous apelin ligands and attenuate PAH in an MCT model (Yang et al., 2017b). Therefore a range of studies provide evidence that exogenously administered apelin and ELA exert a protective effect and support the receptor as a new therapeutic target for peptide and small molecule agonists.

In HF it has been shown in several studies that apelin levels are decreased in human patient plasma (Földes et al., 2003; Chong et al., 2006; Francia et al., 2007), in tissues from a number of rat models (Iwanaga et al., 2006; Koguchi et al., 2012), in mouse doxorubicin-induced cardiotoxicity, and in a dog model (Wang et al., 2013a). Another study in human suggested that apelin plasma levels are only decreased in late-stage HF and are in fact increased in early stage disease (Chen et al., 2003). Such an effect would be unsurprising, given what is known about HF progression, with an often compensatory early stage followed by maladaptation later. However, these results were not corroborated in a study on Dahl salt-sensitive rats in which there was no change in apelin levels in the early stage of the disease but the levels did decrease as expected in later disease (Iwanaga et al., 2006). Overall, these studies support plasma apelin levels as a useful biomarker of disease progression. Importantly, apelin levels can also be modulated in disease and it has been shown that patients fitted with LV assist devices display higher apelin levels in addition to improved disease parameters (Chen et al., 2003; Francia et al., 2007).

Although apelin is highly downregulated, expression of the apelin receptor appears to be less strongly suppressed in these diseases and crucially remains responsive to apelin. In rats induced to develop PAH by hypobaric hypoxia, apelin receptor levels were unchanged (Andersen et al., 2009), whereas in an MCT model, the apelin receptor was downregulated in concert with apelin (Falcão-Pires et al., 2009). In rat models of HF, apelin receptor expression has mostly been found to decrease. This was the case in LV myocytes from hypertensive animals, where infusion of [Pyr^1^]apelin-13 restored some receptor expression (Pang et al., 2014), as well as in the myocardium of an isoproterenol-induced injury model (Jia et al., 2006). Studies using Dahl salt-sensitive hypertensive rats have demonstrated a decrease in apelin receptor expression in the same pattern as observed for the apelin peptide (Iwanaga et al., 2006; Koguchi et al., 2012), whereas one also found no change in the early stage of the disease (Iwanaga et al., 2006), perhaps suggesting that earlier intervention could be more beneficial. Mice induced to exhibit cardiotoxicity with doxorubicin also displayed reductions in apelin receptor expression (Hamada et al., 2015). A study using microembolization-induced HF in dogs showed that the apelin receptor was not decreased compared with controls, although apelin was (Wang et al., 2013a).

#### 2. Clinical Studies in Pulmonary Arterial Hypertension

In a double-blind randomized crossover study, systemic [Pyr^1^]apelin-13 infusion resulted in a reduction in pulmonary vascular resistance and increased cardiac output in PAH patients. Significantly, the effect of the peptide was enhanced in a subgroup of patients who were also receiving a phosphodiesterase type 5 inhibitor (Brash et al., 2018). Although this was an acute study, it provides proof of principle that apelin receptor agonism was beneficial in these patients particularly in improving cardiac output. Secondly, as predicted from in vitro studies, where apelin induces release of vasodilators through apelin receptors present on the endothelium, phosphodiesterase type 5 inhibitors reduced metabolism of nitric oxide. This is important as the current therapy usually includes a phosphodiesterase type 5 inhibitor in combination with an endothelin receptor antagonist. It has previously been demonstrated in vitro in human isolated vessels that [Pyr^1^]apelin-13 acts as a physiologic antagonist to endothelin-1 (Maguire et al., 2009). This provides tantalizing circumstantial evidence that apelin agonism would be of benefit when used in combination with existing therapies.

Additionally, it has been shown in patients fitted with LV assist devices that the apelin receptor is the most upregulated gene, in concert with improved disease parameters (Chen et al., 2003). Together these studies demonstrate that while the apelin receptor may undergo some downregulation in disease, this is susceptible to modulation and its expression is seen to increase alongside positive changes in cardiac parameters.

Given the downregulation of apelin but maintenance of signaling through the receptor, replacement of the downregulated endogenous agonist could be useful therapeutically. One concern with this strategy is that in the diseased state, one would anticipate the endothelium becoming damaged and therefore less responsive to apelin, limiting therapeutic efficacy. However, previous work has shown that infusion of apelin is beneficial in a number of disease models. [Pyr^1^]apelin-13 given intraperitoneally was able to alleviate right ventricle hypertrophy and diastolic dysfunction in the MCT rat model of PAH (Falcão-Pires et al., 2009). It has also been shown that ELA is able to replace the missing endogenous apelin peptide and prevent PAH in an MCT model (Yang et al., 2017b). In several rat models of HF, including isoproterenol-induced (Jia et al., 2006); Dahl salt-sensitive hypertensive (Koguchi et al., 2012); two-kidney, one clip hypertensive (Pang et al., 2014); and left anterior descending coronary artery ligation (Berry et al., 2004; Atluri et al., 2007), apelin induced beneficial changes in the disease state. In mice subjected to transverse aortic constriction to induce a pressure-overload model of HF, [Pyr^1^]apelin-13 administered intraperitoneally in liposomal nanocarriers displayed a longer duration of action and a reduction in LV size and fibrosis compared with mice administered [Pyr^1^]apelin-13 (Serpooshan et al., 2015). Such a result is promising in suggesting that simply by extending the duration of action, rather than the dose of [Pyr^1^]apelin-13 administered, it would be possible to extend the benefits observed in disease. The improvements in rodent models were recapitulated in the microembolism-induced dog model given apelin-13 discussed earlier, with an improvement in ejection fraction and LV systolic parameters observed acutely (Wang et al., 2013a). As well as demonstrating that apelin can be beneficial when infused, studies have also shown that deletion of the apelin receptor and ligand exacerbate disease. In a doxorubicin-induced mouse model of cardiotoxicity, deletion of the apelin receptor led to a more severe reduction in cardiac contractility and accelerated myocardial damage compared with control animals (Hamada et al., 2015). At the same time, apelin knockout mice developed impaired cardiac contractility when aged, or chronic HF when subjected to pressure overload (Kuba et al., 2007). An interesting study in patients has also hinted at beneficial effects of apelin in humans (Boal et al., 2015). This study discussed the idea that patients with higher body mass indices and decompensated HF displayed lower in-hospital mortality, while apelin is also increased in these patients with obesity patients. Using an obese mouse model subjected to ischemia-reperfusion to test this hypothesis, they suggest a mechanism whereby apelin prevents the nuclear translocation of a transcription factor critical to mitochondrial regulation, FOXO3, thus promoting cell survival pathways and consequent cardioprotection. In conclusion, there is strong evidence that the apelin-apelin receptor signaling axis is perturbed in both PAH and HF and that modulation of this system by reintroduction of the downregulated apelin ligand or of ELA could be beneficial.

### E. Diabetes and Metabolic Disease

Type two diabetes mellitus (T2DM) is a common, long-term metabolic disorder characterized by hyperglycemia (high blood glucose) resulting from insulin resistance and relative insulin insufficiency. Insulin resistance describes the stage at which a greater than normal amount of insulin is required to obtain a normal physiologic response to glucose homeostasis. Insulin resistance is not only the main underlying disorder in the pathophysiology of T2DM, but also a well-recognized risk factor for cardiovascular disease (Balkau and Eschwège, 1999; Patel et al., 2016) and has been the focus of therapeutic intervention to date.

Apelin has been highlighted as a naturally occurring peptide which inhibits insulin secretion, decreases glucose levels, increases insulin sensitivity and has a role in the pathogenesis of diabetes related complications.

In vitro studies in both human and murine pancreatic islets have shown that apelin is predominantly expressed in *β*- and *α*-cells, while the apelin receptor is found on INS-1 clonal *β*-cells (Ringström et al., 2010). Selective deletion of the apelin receptor in pancreatic islet *β*-cells of mice resulted in a reduction of islet size, density, and cell mass. These cells showed a decreased insulin secretion in vitro, and the mice displayed decreased glucose tolerance (Li et al., 2006; Han et al., 2015). Insulin exerted direct control on apelin gene expression in adipocytes of humans and rodents and there was a large increase in apelin expression in fat cells of all hyperinsulinemia-associated obesities (Boucher et al., 2005). Both in vitro and in vivo studies have demonstrated that apelin inhibits either glucose- or GLP-1-stimulated insulin secretion (Sorhede Winzell et al., 2005; Guo et al., 2009) and decreases glucose levels in mice after intravenous apelin injection (Dray et al., 2008). Furthermore, apelin reduced insulin resistance during a hyperinsulinemic-euglycemic clamp in mice by stimulating glucose uptake in soleus muscle (Dray et al., 2008). The role of apelin in insulin resistance has also been studied in mice with generalized apelin deficiency; these mice were insulin resistant and the resistance was reversed after exogenous apelin administration (Yue et al., 2010). Further studies in mice have shown that chronic apelin treatment decreases insulin resistance by increasing fatty acid oxidation and mitochondrial biogenesis (Attané et al., 2012).

Finally, in a study to assess whether apelin could be beneficial when administered in a diabetic rat model, it was found that [Pyr^1^]apelin-13 could induce a reduction in plasma insulin and glucose levels with a concurrent reduction in blood pressure (Akcılar et al., 2015a). Long-term treatment with acylated analogs of apelin-13 amide [particularly pGlu(Lys^8^GluPAL)apelin-13] ameliorated diet induced obesity-diabetes in mice and improved lipid profile (O’Harte et al., 2018a). Apelin-13 analogs with improved in vitro plasma stability, potently lowered glucose and insulin levels in vitro (isolated mouse pancreatic islet cells) and in vivo (O’Harte et al., 2018b). These studies, therefore, indicate an important role for the apelin system in the regulation of glucose in diabetes and suggest it could be used potentially as a therapeutic intervention.

There are limited published data studying the relationship between apelin, plasma glucose levels, and insulin sensitivity in humans. Observational human studies have confirmed the presence of increased apelin concentrations in type 2 diabetes mellitus and obesity (Castan-Laurell et al., 2011), and they have raised the possibility of its use as a biomarker (Ma et al., 2014). Studies in patients receiving either metformin alone or in combination with vildagliptin demonstrated that apelin levels were enhanced when compared with controls for those receiving the monotherapy and even more greatly increased when on combinatorial therapy (Fan et al., 2015b). These increases in apelin were seen alongside an improvement in fasting glucose and glycosylated hemoglobin levels, supporting the use of apelin as a marker of improved diabetic control in patients.

Only one published study has measured the metabolic effects of intravenously infused apelin in humans. Gourdy et al. (2018) demonstrated that apelin infused over a 2-hour period during hyperinsulinemic-euglycemic clamping improves insulin sensitivity in overweight men. Meanwhile, unpublished data from our group demonstrated a decrease in blood glucose in nondiabetic volunteers following a 6-minute apelin intravenous infusion (100 nmol/min).

Diabetes is associated with the development of microvascular complications (diabetic nephropathy, neuropathy, and retinopathy) and macrovascular complications (coronary artery disease, peripheral arterial disease, and stroke). The role of apelin and its connection with the etiopathogenesis of diabetes-related complications is being increasingly investigated.

Patients with diabetes can develop proliferative diabetic retinopathy, characterized by fibrovascular proliferation, subsequent bleeding, and retinal detachment (Wu et al., 2017a). Although the exact etiopathogenesis remains unclear (Crawford et al., 2009), apelin may contribute by promoting proliferation and migration of retinal pigment epithelial cells, collagen I expression, and retinal neovascularization (Wu et al., 2017a). Apelin is also coexpressed with VEGF in epiretinal membranes and was found to be elevated in eye vitreous (Wu et al., 2017a).

The apelin receptor is localized to the glomeruli and blood vessels in the kidneys; however, the role of the apelin system in diabetic nephropathy remains controversial. Experiments in rodents showed that apelin has protective effects on the diabetic kidney through antioxidant and anti-inflammatory mechanisms. It has been shown that short-term subcutaneous apelin administration is sufficient to reduce kidney and glomerular hypertrophy, as well as renal inflammation, while in prolonged 6-month treatment, it also improves albuminuria. These protective effects are believed to be by antioxidant mechanisms such as upregulation of antioxidant catalase (Day et al., 2013). Apelin also regulates kidney inflammation by modulating inflammatory response and by inhibiting histone hyperacetylation (Hu et al., 2016). In contrast, some research has indicated that apelin may aggravate the progression of diabetic nephropathy by inducing podocyte dysfunction and abnormal glomerular angiogenesis (Guo et al., 2015; Hu et al., 2016).

Apelin may have a therapeutic role in diabetic cardiomyopathy and is known to be the most potent endogenous inotropic agent (Maguire et al., 2009). It may alleviate microvascular insufficiency through vasodilatation (Maguire et al., 2009) and promoting neovascularization (Hu et al., 2016). Apelin increases vascular density by upregulating the mitochondrial enzyme sirtuin 3, as well as angiopoietins/Tie-2 and VEGF/VEGF receptor 2 expression (Hu et al., 2016). In the heart, apelin also reduces glucose intolerance in obese hyperinsulinemic rodents and increases glucose uptake (Dray et al., 2008).

Overall, these studies suggest that patients with T2DM may benefit from apelin administration by improving insulin sensitivity, increasing cardiac output (through direct heart stimulation) and enhancing blood flow (through blood vessel dilatation). Nevertheless, the role of the apelin system in relation to diabetes and its complications is still unclear and further investigation is required.

### F. Gastrointestinal Function

Given that apelin was first identified in bovine stomach extracts and is implicated in metabolic disease, it is not surprising that studies have looked for a direct role of apelin in the gut. Apelin mRNA is present in the gastrointestinal tract of the adult rat, with highest levels observed in the stomach fundus, followed by ileum, duodenum, jejunum, and colon (Wang et al., 2004). Interestingly, the same study suggests apelin mRNA levels are significantly higher in fetal and postnatal rat stomachs than in adult stomachs. However, immunohistochemistry suggests the peptide is not present until 20 days postpartum, around the time of weaning. A further study confirmed this and also demonstrated localization of the apelin receptor to the stomach epithelium of developing rats (Wang et al., 2009). The current consensus suggests an inhibitory mechanism that prevents translation of apelin mRNA in rat neonates until they transition from milk to solid food. The authors hypothesize that apelin is delivered in maternal milk and inhibits its own translation, although this is yet to be confirmed. Apelin mRNA is highly expressed in pregnant rat mammary glands and bovine colostrum and is still detectable in commercial bovine milk (Habata et al., 1999; Hosoya et al., 2000; Mesmin et al., 2011), possibly supporting a dietary role in neonatal mammals.

The apelin peptide is localized in vesicle-like structures adjacent to the lumen of gastric glands in the stomach, suggesting luminal secretion (Wang et al., 2004). Apelin is present in the mouse gut lumen and secretion of the [Pyr^1^]apelin-13 isoform is stimulated by glucose ex vivo and in vivo. Once luminal, [Pyr^1^]apelin-13 acts to significantly reduce levels of sodium glucose transporter 1 and increase glucose transporter 2 in the brush border of enterocytes. Overall, this results in an increase in intestinal transepithelial glucose transport from the lumen to the bloodstream. Furthermore, [Pyr^1^]apelin-13 administered by oral gavage increases hepatoportal plasma glucose in mice, while glycemia induced by an oral glucose load is effectively blocked by oral administration of the apelin receptor antagonist MM54 (Dray et al., 2013). The authors propose a gut glucose-apelin cycle, where glucose induces luminal secretion of apelin from intestinal cells before apelin, in turn, facilitates further absorption of glucose. However, such a hypothesis seems to contradict the previously observed hypoglycemic action of intravenously administered apelin-13 (Dray et al., 2008) (see section XI.E). Why apelin increases blood glucose via absorption from the intestinal lumen yet lowers it systemically remains unclear.

In addition to lumenal regulation of glucose absorption, several in vitro studies using a mouse enteroendocrine cell line (STC-1) have demonstrated that apelin-12 and apelin-13 stimulate release of cholecystokinin (CCK). Interestingly, apelin-12 is more potent than apelin-13, while apelin-36 does not stimulate secretion of CCK in STC-1 cells (Wang et al., 2004; Flemström et al., 2011; Wattez et al., 2013). Moreover, in the rat in vivo, intravenous administration of apelin-13 induces luminal secretion of CCK (Wattez et al., 2013) and intra-arterial infusion of apelin-13 induces secretion of bicarbonate in the duodenum that is blocked by the CCK selective antagonist devazepide (Flemström et al., 2011). Furthermore, CCK is secreted from isolated rat intestinal mucosal cells following apelin-12 (Flemström et al., 2011) or apelin-13 (Wattez et al., 2013) treatment. While CCK secretion was shown to be induced by systemic administration of apelin in vivo, these authors hypothesize that apelin in the gut lumen, either from the glucose-apelin cycle or dietary intake, may contribute to CCK release. Apelin-13 also induces secretion of glucagon-like peptide 1 (GLP-1) in STC-1 cells and in vivo in anesthetized rats when administered intravenously (Wattez et al., 2013). On the other hand, [Pyr^1^]apelin-13 inhibits insulin secretion in rat insulinoma cells (INS-1) (Guo et al., 2009), while intravenous administration of apelin-36 inhibits insulin secretion in mice (Sörhede Winzell et al., 2005) (see section XI.E). How and why apelin induces secretion of the incretin GLP-1 yet reduces insulin secretion is currently unresolved.

Apelin was also recently shown to undergo transcytosis from the lumen to intraduodenal structures, such as neuronal plexuses. Here, the apelin receptor is present in excitatory choline acetyl transferase (ChAT) expressing motor neurons and inhibitory neuronal nitric oxide synthase expressing neurons of the enteric nervous system (Fournel et al., 2017). In this setting, a “physiologic switch” was proposed where apelin-13 at 100 nM stimulates only ChAT neuron firing, resulting in increased duodenal contractility and increased glucose absorption. However, 1 *μ*M apelin-13 stimulates both ChAT and neuronal nitric oxide synthase neurons, inducing increased duodenal nitric oxide and no overall change in duodenal contractility or glucose absorption from baseline. This is reminiscent of the glucose-apelin cycle study where 1 *μ*M [Pyr^1^]apelin-13 induced weaker downregulation of sodium glucose transporter 1 than a 100 nM concentration (Dray et al., 2013), potentially indicating a similar physiologic switch. Importantly, chronic oral administration of 1 *μ*M apelin-13 reverses duodenal hypercontractility, lowers glucose absorption, and improves insulin sensitivity in obese/diabetic mice (Fournel et al., 2017), indicating a potential for apelin as an oral therapeutic in metabolic disease.

Apelin is also implicated in colitis and repair of colonic epithelium. In sodium dextran sulfate (DSS)-induced rat and mouse models of colitis, colonic apelin mRNA increased during inflammation as well as the tissue repair stage following the DSS treatment. Immunostaining revealed a similar pattern for levels of the peptide where apelin immunoreactivity increased in the surface epithelium, in epithelial cells of tubular glands, and in the stem cell regions at the bases of the glands in animals treated with DSS. The same study showed epithelial apelin immunoreactivity is also higher in colonic tissue from human patients with ulcerative colitis or Crohn’s disease than in control histologic samples. Finally, subcutaneous [Pyr^1^]apelin-13 administration three times a day in mice pretreated with DSS stimulates colonic epithelial cell proliferation. The study concludes that increased apelin in early and repair phases of colitis may facilitate recovery of colonic tissue and that exogenous administration of the peptide may be beneficial in human bowel disease (Han et al., 2007).

Evidence suggests that apelin signaling may play an important role in gastrointestinal physiology and pathophysiology, although the precise functions remain incompletely understood. On top of this, the role of the second endogenous apelin receptor ligand, ELA, is wholly unexplored, either exogenously or endogenously in the gastrointestinal tract, and it will be of great interest to see what complexities this peptide contributes to the field.

### G. Human Immunodeficiency Virus/Simian Immunodeficiency Virus Coreceptor

Human immunodeficiency viruses (HIV) and Simian immunodeficiency viruses (SIV) generally use the CD4 receptor and a coreceptor, usually one of the chemokine receptors CCR5 or CXCR4, to infect host cells. However, the apelin receptor (which displays some structural similarities to CXCR) can also perform this function for a number of HIV-1 and SIV strains (Choe et al., 1998; Edinger et al., 1998; Zhang et al., 1998; Puffer et al., 2000) and this can be blocked by apelin (Cayabyab et al., 2000; Puffer et al., 2000; Zou et al., 2000). Key interactions occurred through the first 20 N-terminal amino acids of the apelin receptor and the gp120 HIV-1 viral protein. Apelin blocked this by interacting with the second 10 residues of the N terminus (Zhou et al., 2003a). Since these early experiments, there have been few follow-up studies and the relevance and implications of apelin receptor involvement in HIV-1/SIV infection have not been addressed. Intriguingly, interplay between the apelin receptor and CXCR4 has a role in vascular maturation in murine retinas, whereby apelin signaling upregulates transcription of microRNA 139-5p, in turn downregulating CXCR4 to promote healthy retinal vascularization (Papangeli et al., 2016).

### H. Fluid Homeostasis

Apelin has been shown to have roles in the uptake and retention of fluid as reviewed by Flahault et al. (2017). These responses were modest and appear mostly to be through interactions with regions of the central nervous system associated with arginine-vasopressin. Apelin receptor knockout mice drank less than wild-type mice, also although the volume and osmolality of urine excreted did not differ. Furthermore, although wild-type mice showed reduced urine volume and increased osmolality following 24-hour water deprivation, apelin receptor knockout mice did not (Roberts et al., 2009). In rats injected intracerebroventricularly with [Pyr^1^]apelin-13, a dose-dependent increase in drinking behavior and water uptake was observed (Taheri et al., 2002). It was also shown more recently that apelin-17 can directly affect the collecting ducts in the kidney to counteract the antidiuretic effects of vasopressin, thus enhancing the diuretic effects of apelin and the complexity of apelin-vasopressin interactions (Hus-Citharel et al., 2008, 2014).

### I. Age-Associated Sarcopenia

The apelin receptor was recently implicated in age-associated sarcopenia, which is a degenerative disease involving loss of skeletal muscle mass and strength, ultimately resulting in disability and, frequently, hospitalization of the elderly. A single study (Vinel et al., 2018) has investigated this and, interestingly, found that apelin levels are negatively correlated with age in rodents and humans. Furthermore, restoration of apelin signaling had a positive effect on muscle function in ageing mice deficient in either apelin or its receptor. There is evidence to suggest that apelin peptide is endogenously secreted from skeletal muscle upon contraction to induce anti-inflammatory pathways, mitochondriogenesis, and autophagy to improve muscle function. This may provide an explanation for the beneficial effects of exercise on decreasing age-associated sarcopenia in elderly individuals. Overall, apelin has potential for use as a biomarker and therapeutic strategy in this disease.

## XII. Elabela/Toddler Physiology and Pathophysiology

### A. Developmental Roles

ELA was first discovered as a critical developmental peptide in zebrafish cardiogenesis (Chng et al., 2013; Pauli et al., 2014), an important role that is supported by the high degree of conservation at the functional C-terminal end of the peptide. This effect is mediated through the apelin receptor and may occur through an enhancement of nodal/TGF*β* signaling. In fact, nodal elevation in aplnra/b double knockout zebrafish embryos is sufficient to rescue them from the resulting cardiac differentiation defects (Deshwar et al., 2016). Following its identification, it was not initially clear whether ELA was involved in other developmental mechanisms and, consequently, a number of studies set out to investigate this.

In experiments also studying zebrafish embryos, Helker et al., (2015) demonstrated that ELA was important for angioblast migration during the formation of large axial vessels, the dorsal aorta, and cardinal vein. Crucially, this was through the apelin receptor with knockout embryos unable to form the vessels correctly. Additionally, wild-type angioblasts injected into a knockout background embryo were able to restore function, showing that these cells were responding to secreted ELA.

In mammalian development, ELA has been studied in human embryonic stem cells (ECSs; Ho et al., 2015) and mouse ESCs (Li et al., 2015), where it functions to maintain self-renewal. Both of these studies reported that neither human nor mouse ESCs express the apelin receptor. However, in both cases, they do demonstrate ELA expression. Ho et al., (2015) reported that a loss of ELA by shRNA knockdown leads to a loss of morphology and an inability to form teratomas, a characteristic trait of stem cells. This could be rescued by exogenously applied ELA, perhaps suggesting an alternative receptor in this cell type. Li et al. (2015), meanwhile, found that ELA was a downstream target of p53 whose knock down could prevent DNA damage-induced apoptosis in mouse ESCs. They performed a BLAST search to identify potential additional targets of ELA with similar sequences to the apelin receptor but claim not to have identified any. They instead suggested that ELA RNA is involved in a feedback loop with p53 in mouse ESCs to regulate DNA damage-induced apoptosis and demonstrated that the coding region of ELA is dispensable to function in support of this. Recently, it was shown that the ELA-apelin receptor axis plays an important role in coronary artery development in the mouse heart (Sharma et al., 2017), supporting zebrafish studies and the vasculature defects observed in knockout organisms. In this study, Sharma et al. (2017) genetically labeled apelin receptor expressing cells and found that these cells were found in sinus venosus and not endocardial coronary progenitors. In both homo- and heterozygous apelin receptor mutants, the sinus venosus coronary progenitors failed to migrate properly to form vasculature structures in the heart, but this failure could be compensated for by enhanced endocardial progenitor proliferation. ELA mutant hearts phenocopied the receptor mutants but apelin mutants did not, supporting ELA as the important signaling peptide in this pathway.

Intriguingly, ELA also has an effect on development via maternal expression. In both mice and humans, the chorionic ectoderm and syncytiotrophoblast of the developing placenta express high levels of ELA, where it promotes heathy placental development and angiogenesis. In pregnant mice, ELA knockout resulted in defective placenta, causing preeclampsia, a gestational hypertensive syndrome highly implicated in fetal and maternal morbidity and mortality (Ho et al., 2017).

### B. Adult Cardiovascular Roles

In addition to being present in various mammalian cell types including human pluripotent stem cells, Wang et al., (2015b) showed that ELA was able to perform functions in adult cell types and tissues. They demonstrated that ELA could induce angiogenesis of human umbilical vein endothelial cells in vitro in Geltrex-coated wells and that this was abolished if apelin receptor was knocked down. They also reported that ELA could induce relaxation in mouse aortic blood vessels. Since then, further studies have identified ELA as a positive inotrope and vasodilatory agent ex vivo (Perjés et al., 2016) and in vivo in rats (Murza et al., 2016; Yang et al., 2017b). The study by Yang et al. (2017b) also looked at the ability of ELA peptides to bind to the apelin receptor in homogenates of human LV and to activate signaling pathways in cell-based assays expressing the human receptor. This study went on to demonstrate that ELA is present in both large and small diameter human blood vessels, as well as in human plasma.

### C. Disease and the Possibility of a Second Receptor for Elabela/Toddler

Perhaps most interestingly, the study by Yang et al. (2017b) showed that ELA was downregulated in plasma serum, pulmonary arterial endothelial cells, and pulmonary microvascular endothelial cells from patients with PAH, raising the possibility that ELA is not only functional in the adult human but also altered in the diseased state. They went on to demonstrate that ELA was able to attenuate the onset of PAH in a MCT rat model. A number of other studies have also looked at the ability of ELA to modify disease in animal models. Chen et al. (2017a) demonstrated that both ELA-32 and ELA-11 could protect against renal ischemia-reperfusion injury, while Schreiber et al. (2017) showed that adenovirally delivered ELA could protect against kidney damage in a Dahl salt-sensitive rat model. However, what is still unclear is whether ELA mediates these protective effects through the apelin receptor or through a distinct receptor. Several studies have produced contradictory evidence regarding this.

Chen et al. (2017a) used siRNA knockdown of the apelin receptor in NRK-52E cells and did not observe any changes in cell viability either in normoxic or hypoxic conditions. Meanwhile, Ho et al. (2017) looked at ELA and apelin knockout in pregnant mice. They showed that ELA, but not apelin knockouts, displayed preeclampsia-like symptoms and that infusion of ELA could alleviate them. They also demonstrated that ELA knockout mice placentas were not rescued by apelin but were by ELA infusion, again suggesting differences in signaling pathways. They conclude that the preeclampsia alleviation is likely achieved through apelin receptor signaling in endothelial cells. They also do not rule out a possible contribution from additional unidentified ELA receptors. On the contrary, Sato et al. (2017) suggested that administration of ELA peptide could protect against cardiac dysfunction, hypertrophy, and fibrosis in pressure overload mice. Importantly, apelin receptor knockout mice did not respond to ELA administration, demonstrating that the protection was mediated by the ELA-apelin receptor axis. These studies raise important questions about whether such a second receptor for ELA exists. If this receptor was another GPCR, it would be remarkable that it should be selective between apelin and ELA. Indeed, while many peptides bind to multiple GPCRs, it is rare to have a GPCR with multiple ligands and would be even more so if they should have such diversity in structure to enable binding to additional targets. It is likely that, as many ligands do, apelin and ELA have different signaling profiles in different tissues/organs (as well as between cell and animal models), and this could explain discrepancies in ELA and apelin knockouts; however, it would not be able to explain the differences in outcomes observed when the receptor is knocked out. There has been some evidence, as mentioned previously, of ELA RNA having a role in development before the apelin receptor is expressed (Li et al., 2015) and it could be that such mechanisms are maintained to adulthood. The interactions of apelin, ELA, and the apelin receptor in such conditions, therefore, warrant further investigation.

## XIII. Human Polymorphisms

### A. Apelin Receptor

Single nucleotide polymorphisms (SNPs) in the apelin receptor have been reported in a number of populations. The majority occur outside the coding regions of the gene because of its critical importance in development. However, they may be predictive of outcomes either through linkage with other SNPs or through affecting transcription factor binding. Although the nucleotide substitutions in this review are given for the forward strand of the gene as identified in the NCBI SNP database and with the most common ancestral allele listed first, it should be noted that many of them have alternative aliases that may refer to the reverse orientation. When these occur prominently in the literature, they have also been used but with the forward allele put in brackets for clarification.

The rs948847 (A445C) SNP (G/T) occurs in a coding region of the gene but predicts no change in amino acid (glycine). In Italian populations it has been shown to have no relation to either heart failure related events (Sarzani et al., 2007) or coronary artery disease (CAD) (Falcone et al., 2012). Similarly, Akcılar et al. (2015b) demonstrated no difference in allele frequencies between patients with CAD and controls. However, they found that carriers of the CC (GG) genotype had greater weights and higher systolic and diastolic blood pressures. Soualmia et al. (2017) in a Tunisian population found no association with diabetic retinopathy but found that CC (GG) bearers had higher total cholesterol levels than those with the AA (TT) genotype. These studies suggest that although the C (G) allele may not be able to predict disease in these populations, it could contribute to risk factors for cardiovascular disease. In fact, one study by Hata et al. (2007) in Japan found an association with brain infarction events and went on to demonstrate that only the A (T) allele may be able to form a DNA-protein complex, perhaps suggesting a mechanism for the difference, although no transcription factor was predicted to bind.

The rs9943582 (G154A) SNP (C/T) occurs in the 5′ untranslated region of the gene. In a Han Chinese population it has not shown association with the age of onset or clinical outcomes of ischemic stroke (Zhang et al., 2017). However, in a meta-analysis conducted by Chen et al. (2017c), it was reported that the T allele carried a 5.2% increased risk for CAD compared with the C allele. Hata et al. (2007) suggested that the G (C) allele of this SNP had an association with brain infarction in a Japanese population and that this was caused by higher binding of the transcription factor, sp1, leading to greater enhancer activity. Follow-up studies by the same group have focused on the relationship of the G (C) allele with ischemic stroke and have suggested that this allele leads to higher apelin receptor mRNA levels due to the higher binding affinity of sp1 (Hata et al., 2011). They went on to suggest that the G (C) allele was more likely to trigger changes in blood pressure and atherosclerosis with a connection to stroke propensity (Hata et al., 2011). Hinohara et al. (2009) in a Japanese and Korean study reported no association of the polymorphism to CAD or coronary atherosclerosis. Similarly, Jin et al. (2012) found no association in single-locus analysis in a population of Chinese with hypertension. However, they found a sex-specific female association with CAD when analyzed in concert with rs7119375. Huang et al. (2016a) also found no differences in allele distribution between hypertensive and control Chinese patients. In a Han Chinese population of CAD patients, Wang et al. (2015a) suggest that the A (T) allele was negatively associated with left ventricular (LV) systolic function, LV end-diastolic diameter, left atrial diameter, and LV ejection fraction. Interestingly, these findings are contrary to the reports by Hata et al. (2007) suggesting the risk allele as the G (C) variant.

The rs7119375 SNP (G/A) is near the 5′ untranslated terminus and has been reported in a number of Chinese populations. As discussed earlier, it has been shown to have an association with CAD in female patients with hypertension when analyzed with rs9943582 (Jin et al., 2012). It has also been shown in Han Chinese populations to have an association with essential hypertension, body mass index, and the onset age of hypertension when analyzed with rs10501367 (C/T) (Li et al., 2009), although this may be sex dependent or depend on the haplotype of other SNPs identified in the study (Niu et al., 2010). Huang et al. (2016a), in contrast, found no significant differences between hypertensive patients and controls. This could reflect the sex differences suggested earlier, and Li et al. (2016) demonstrated that the AA genotype was associated with higher systolic blood pressures than the GG combination in both hypertensive and control Chinese female patients, with carriers of the A allele having a 1.8 times greater risk of developing hypertension.

The rs11544374 (G212A) SNP (G/A) also occurs in the 5′ untranslated region. In a Southern Italian population, Sarzani et al. (2007) studied the progression of patients with idiopathic dilated cardiomyopathy compared with controls and suggested a lower risk of heart failure-related events in patients bearing the A allele. Falcone et al. (2012) again in an Italian population studied association with CAD. They found no relationship, but did find evidence of an association with hypertension in CAD patients possessing the G allele. In an Indian study, Mishra et al. (2015) looked at association with high-altitude pulmonary edema (HAPE) by comparing patients with HAPE, HAPE-free sojourners, and healthy highland natives. They found an overrepresentation of the A allele in patients with HAPE and, most interestingly, found that it was associated with reduced apelin-13 levels in patients with HAPE and healthy highland natives. This is contrary to the findings of Kotanidou et al. (2015) in Greek children and adolescents. They found that apelin was reduced in youngsters with obesity but that the A allele was associated with higher apelin levels in this instance.

Several SNPs have been studied through multiple loci analyses, some of which have already been discussed. The following, rs721608 (A/G), rs746886 (G/A), and rs2282623 (C/T), are SNPs in the 3′-untranslated region. These were studied in a Han Chinese population, and it was found that rs746886 and rs2282623 demonstrated associations with diastolic and mean arterial blood pressure responses to low-sodium intervention. It was then shown that the A-T(A)-T haplotype of the three SNPs was related to decreased diastolic blood pressure and mean arterial blood pressure responses to low sodium, while the G-C(G)-C haplotype was associated with increased systolic blood pressure and mean arterial blood pressure responses to high sodium (Zhao et al., 2010). The rs2282623 SNP was also studied by Mishra et al. (2015) and, like with rs11544374, they found a difference between patients with HAPE, HAPE-free sojourners, and healthy highland natives with an overrepresentation of the G (C) allele in this case.

### B. Apelin

In addition to polymorphisms in the apelin receptor, a number of SNPs have been identified in the apelin peptide and studied in similar diseases. These all occur in noncoding regions of the gene, and this is consistent with the high degree of amino acid conservation of the peptide between species.

The rs3761581 SNP (G/T) is located in a suggested promoter region of the 5′-untranslated region, and many studies refer to it as an A/C substitution according to the reverse strand orientation. In Chinese populations, it has been found that there is a significant association with hypertension in men but not women (Niu et al., 2010; Huang et al., 2016a) but that the A (T) allele increased the risk of hypertension regardless of sex (Huang et al., 2016a). Meanwhile, Zhu et al. (2015) suggested an association with combined hypertension and renal artery stenosis. In Indian populations, Gupta et al. (2016) looked at associations in essential hypertension and acute coronary syndrome and found that the G allele was elevated in acute coronary syndrome, although there was not a significant association. Mishra et al. (2015) also looked in Indian populations and support the G allele as the risk allele, finding a significant elevation in patients with HAPE compared with HAPE-free sojourners or healthy highland natives.

The rs56204867 (T-1860C) SNP (A/G) occurs in a promoter region and has largely been studied in Chinese populations. Li et al. (2009) found that in combination with the rs3761581 SNP, the haplotypes T(A)-A(T) and C(G)-C(G), respectively, were significantly associated with hypertension, increased BMI, and earlier onset age of hypertension. Huang et al. (2016a) suggested the C (G) allele increased the risk of hypertension regardless of sex, although only men showed a significant difference between patients with hypertension and controls. In contrast, Niu et al. (2010) found that it was not associated with hypertension as a single locus. This was followed up by Jin et al. (2012), who demonstrated no association with CAD as a single locus but a haplotype G-A(T) association with rs56204867and rs3761581, respectively. In a statin drug treatment study, Jia et al. (2015) found that women with a TT (AA) genotype had a greater systolic blood pressure reduction after 24 weeks of treatment compared with patients with the C (G) allele. In a Turkish population, it was found that the C (G) allele was higher in patients with CAD than controls and that patients with the CC (GG) genotype had lower apelin levels than those with the TT (AA) genotype (Akcılar et al., 2015c).

Liao et al. (2011) found a significant association of the rs3115757 SNP (G/C) with BMI and waist circumference in Chinese women but not men. They compared the CG and GG genotype to the CC genotype and found that the latter was more likely to have higher BMI and waist circumference. In adipocytes from women of the CC genotypes only, apelin mRNA and protein concentrations were higher in cells treated with high glucose plus insulin than in those with normal glucose. In contrast, in Egyptian women it has been shown that the GG genotype is associated with higher BMI and waist circumference and a 12 times higher risk of developing obesity; these individuals also showed higher insulin resistance (Aboouf et al., 2015). Further studies in Chinese populations have suggested that the C allele might have detrimental effects on arterial stiffness with rank order CC>CG>GG (Liao et al., 2015) and carries an increased risk of hypertension regardless of sex (Huang et al., 2016a). Whereas in Indian patients with HAPE, there is an overrepresentation of the G allele compared with HAPE-free sojourners or healthy highland natives (Mishra et al., 2015). The study by Liao et al. (2015) has also implicated the rs3115758 SNP (T/G), which is in complete linkage disequilibrium with rs3115757. They identified it in the binding site of miRNA-765 and -650 and showed that the C (G) allele is associated with arterial stiffness in women but not men. As a mechanism they suggested that the C (G) allele increases the binding of miRNA-765, which leads to reduced apelin expression. Another study implicated the TT genotype as a risk factor for CAD in a Turkish population (Pakizeh et al., 2015). This last study also implicated the AA genotype of the rs3115759 SNP (A/G) in Turkish CAD patients.

The rs2235312 SNP (G/A) has been studied in Indian populations, but appears in the literature with the T/C notation. Mishra et al. (2015) found an overrepresentation of the T (A) allele in patients with HAPE compared with HAPE-free sojourners or healthy highland natives in a genome-wide association study. They showed that in patients with HAPE and healthy highland natives, this T (A) allele was associated with decreased apelin-13 and nitrate levels. In a study in a North Indian population, Gupta et al. (2016) found that the SNP was not associated with essential hypertension and acute coronary syndrome.

In type II diabetes, the rs2281068 SNP (C/T) has been shown to have a significant correlation in a Chinese population (Zheng et al., 2016). Meanwhile, the rs2235306 SNP (T/C) has been nominally associated with fasting glucose levels in male Han Chinese subjects with normal glucose association but not with type II diabetes (Zhang et al., 2009). This latter polymorphism has demonstrated a significant association with diastolic BP responses to potassium supplementation (60 mmol/day) in women, with the response following the genotypic rank order TT>TC>CC (Zhao et al., 2010).

## XIV. Knockout Mouse Models

As discussed earlier, the second peptide at the apelin receptor, now identified as ELA, was predicted due to the discrepancies observed between embryonic knockouts of apelin and its receptor (Charo et al., 2009, Table 7). Knockouts of the receptor in mice caused prenatal mortality (Ishida et al., 2004; Charo et al., 2009; Roberts et al., 2009; Scimia et al., 2012; Kang et al., 2013) with a failure in cardiac development as the cause (Kang et al., 2013). Furthermore, neonates that survived to later embryonic stages displayed incomplete vascular maturation, in part due to a deficiency in vascular smooth muscle cells, as well as incomplete ventricular wall development (Kang et al., 2013). Those surviving to adulthood have been shown to display decreased contractile function in the heart, which translates to a reduced exercise capacity (Charo et al., 2009). Interestingly, they also displayed a reduced propensity to develop HF in response to pressure overload (Scimia et al., 2012). In contrast, apelin peptide knockouts had normal heart development (Kidoya et al., 2008; Charo et al., 2009) and were born in Mendelian ratio. They also displayed defects in contractility and exercise tolerance (Charo et al., 2009) and were at a greater risk from age-related and pressure overload-induced HF (Kuba et al., 2007).

**TABLE 7:** Phenotypes observed in apelin, apelin receptor, and apela knockout mouse models The lack of similarity between apelin and apelin receptor knock-out mice prompted the suggestion that there might be another ligand at the receptor. Apela knock-outs largely phenocopy the receptor knock-outs, supporting the idea that ELA is the missing endogenous ligand.

Apelin Knockout Mice	Apelin Receptor Knockout Mice	Apela Mutant Mice
Mendelian birth ratio (Kidoya et al., 2008; Charo et al., 2009)	Loss of homozygous mice (Ishida et al., 2004; Charo et al., 2009; Roberts et al., 2009; Scimia et al., 2012; Kang et al., 2013)	Loss of homozygous mice (Freyer et al., 2017; Ho et al., 2017)
Normal heart morphology (Kuba et al., 2007; Kidoya et al., 2008; Charo et al., 2009)	Severe cardiac and vascular developmental defects (Kang et al., 2013)	Severe cardiac and vascular developmental defects (Freyer et al., 2017; Ho et al., 2017)
Normal blood pressure (Charo et al., 2009)	Normal blood pressure (Ishida et al., 2004; Charo et al., 2009)	Pre-eclampsia (Ho et al., 2017)
Modest decrease in basal cardiac contractility (Charo et al., 2009)	Modest decrease in basal cardiac contractility (Charo et al., 2009)	?
Marked decrease in exercise capacity (Charo et al., 2009)	Marked decrease in exercise capacity (Charo et al., 2009)	?
Severe heart failure in response to pressure overload (Kuba et al., 2007)	Markedly reduced heart failure in response to pressure overload (Scimia et al., 2012)	?

In zebrafish, ELA knockouts phenocopied apelin receptor mutations (Chng et al., 2013; Pauli et al., 2014) and it was this, among other observations, that aided in its identification as an endogenous apelin receptor ligand. It is only very recently that mouse ELA mutants have been produced (Freyer et al., 2017; Ho et al., 2017). The knockout was embryonically lethal, in agreement with its role as a critical developmental signaling molecule. The likely cause of death was misregulation of hematopoietic progenitors at late gastrulation stages, leading to cardiac and vascular defects, thus mimicking some of the observations of Kang et al., (2013). Apelin and ELA double knockouts did not further increase embryonic lethality, supporting the hypothesis that in mammalian systems it is ELA, and not apelin, that plays the crucial developmental role (Freyer et al., 2017). However, the authors suggest that some discrepancies, particularly with regards to survival rates between ELA and apelin receptor knockouts, are suggestive of independent actions of the apelin receptor either through alternative ligands or potential stretch-mediated responses.

Therefore, although it seems evident that ELA plays a critical role in the early development of the cardiovascular system through the apelin receptor, it remains to be seen whether the apelin receptor may have additional developmental roles. Furthermore, generation of an inducible ELA knockout would be interesting by allowing further exploration of its roles in the adult mammalian system. Recent studies have illustrated activity in the adult cardiovascular system, as well as changes in ELA expression in the diseased state (Yang et al., 2017b). Given that the apelin peptide mutant shares some similarities with the adult apelin receptor mutants (Kuba et al., 2007), it is tempting to propose a dichotomous situation with ELA as the key developmental ligand through the apelin receptor. Apelin may function as the key adult signaling peptide, where it is widely expressed throughout mammalian tissues, reflecting its presence within endothelial cells. In contrast, the distribution of ELA is more restricted and may function in specific organs such as the kidney. Such a reductionist view is likely too simplistic, and further exploration of the roles of ELA in the adult system will help to shed light on the possible interplay between the two endogenous apelin receptor agonists in health and disease.

## XV. Conclusions and Perspectives

The field of apelin/ELA research is rapidly expanding, with over 2000 publications with ∼40,000 citations. Key questions remain. It is intriguing to know why two peptide families with only limited sequence similarity have evolved to signal through the same receptor. The majority of peptide families target at least two GPCR subtypes. The importance of the spatiotemporal signaling pathway in cardiovascular development is clear (Chng et al., 2013; Pauli et al., 2014), with loss of ELA causing profound heart defects but the relative contributions of apelin and ELA in adults remains to be resolved. Novel ligands based on ELA structure-activity are beginning to be discovered, and these may enable the action of the two endogenous pathways at the same receptor to be dissected (Murza et al., 2016). The apelin receptor has been shown to be tractable to discovering both peptide and small molecule ligands biased toward activating the G protein pathway to cause the desired vasodilatation and increase in cardiac output without desensitizing the receptor. Importantly, the efficacy of such compounds has been shown in the clinic using volunteers (Brame et al., 2015), one of few GPCR systems to be tested in human studies. Efficacy now needs to be demonstrated in patients, where loss of apelin signaling has been observed in conditions such as PAH or in metabolic disorders such as diabetes where substantial evidence has been obtained in animal models. To date only a few ligands targeting GPCRs have been identified as biased (Wootten et al., 2018). The concept of biased agonism can potentially revolutionize understanding of the fundamental biology of GPCR cell signaling. Intriguingly, the apelin pathway has been implicated in cancer, and apelin ligands have been shown to be efficacious in animal models of the most common brain tumor, glioblastoma. Further studies are needed to exploit this new target, particularly as current treatments lack efficacy. New pharmacological tools need to be developed, especially antagonists with better pharmacokinetics for in vivo use, which is critical for the field to advance. This may also include monoclonal antibodies targeting the receptor (Hutchings et al., 2017). Methods using mass spectrometry are being developed to identify and quantify apelin and ELA isoforms in vivo and in tissues. The N terminus of the apelin receptor was truncated (Ma et al., 2017) to obtain initial X-ray crystallographic data and remains an area for further structural analysis, potentially using new techniques such as cryoelectron microscopy (Draper-Joyce et al., 2018). Finally, there are still over 50 orphan Class A GPCRs, where endogenous ligands remain to be discovered (Davenport et al., 2013). About one-half have been predicted from structural motifs to be activated by peptides. The discovery of ELA, from a region previously classified as noncoding for proteins (Pauli et al., 2015), provides compelling support for further analysis of the human genome to identify the remaining endogenous peptide ligands for orphan GPCRs.

## Abbreviations

ACE2: angiotensin converting enzyme 2
AlbudAb: anti-serum albumin domain antibody
APELA: apelin early endogenous ligand
APLNR: apelin receptor
AT_1_: angiotensin II type 1 receptor
BMI: body mass index
CAD: coronary artery disease
CCK: cholecystokinin
ChAT: choline acetyl transferase
CXCR4: chemokine receptor type 4
DSS: sodium dextran sulfate
ELA: Elabela/Toddler
ESC: embryonic stem cell
GLP-1: glucagon-like peptide 1
GPCR: G protein-coupled receptor
HAPE: high altitude pulmonary edema
HF: heart failure
HGNC: Human Genome Organisation Gene Nomenclature Committee
HIV: human immunodeficiency viruses
IP_3_: inositol triphosphate
LV: left ventricle
MCT: monocrotaline
NC-IUPHAR: The International Union of Basic and Clinical Pharmacology Committee on Receptor Nomeclature and Drug Classification
PAH: pulmonary arterial hypertension
PEG: polyethylene glycol
PKC: protein kinase C
SIV: Simian immunodeficiency viruses
SNP: single nucleotide polymorphism
STAT: signal transducer and activator of transcription proteins
T2DM: type 2 diabetes mellitus
TGF*β*: transforming growth factor *β*
VEGF: vascular endothelial growth factor
